# The BDNF rs6265 Polymorphism is a Modifier of Cardiomyocyte Contractility and Dilated Cardiomyopathy

**DOI:** 10.3390/ijms21207466

**Published:** 2020-10-10

**Authors:** Frank J. Raucci, Anand Prakash Singh, Jonathan Soslow, Larry W. Markham, Lin Zhong, Wejdan Aljafar, Natasja Lessiohadi, Cassandra P. Awgulewitsch, Prachi Umbarkar, Qinkun Zhang, Presley L. Cannon, Maciej Buchowski, Joseph T. Roland, Erica J. Carrier, William B. Burnette, Antonis K. Hatzopoulos, Hind Lal, Cristi L. Galindo

**Affiliations:** 1 Thomas P. Graham Division of Pediatric Cardiology, Vanderbilt University Medical Center, Nashville, TN 37232, USA; frank.raucci@vumc.org (F.J.R.J.); jonathan.h.soslow@vumc.org (J.S.); 2 Division of Pediatric Cardiology, Department of Pediatrics, Children’s Hospital of Richmond at Virginia Commonwealth University, Richmond, VA 23219, USA; 3 Division of Cardiovascular Disease, Department of Medicine, University of Alabama Birmingham School of Medicine, Birmingham, AL 35233, USA; apsingh@uabmc.edu (A.P.S.); pumbarkar@uabmc.edu (P.U.); qzhang@uabmc.edu (Q.Z.); hindlal@uabmc.edu (H.L.); 4 Division of Cardiology, Department of Pediatrics, Riley Hospital for Children at Indiana University Health, Indianapolis, IN 46202, USA; lwmarkha@iu.edu; 5 Division of Cardiovascular Medicine, Vanderbilt University Medical Center, Nashville, TN 37232, USA; lin.zhong@vumc.org (L.Z.); wejdanaljafar@gmail.com (W.A.); Natasjalessiohadi@gmail.com (N.L.); cassandra.p.awgulewitsch@vanderbilt.edu (C.P.A.); pcannon18@outlook.com (P.L.C.); joseph.t.roland@vumc.org (J.T.R.); erica.carrier@vumc.org (E.J.C.); antonis.hatzopoulos@vumc.org (A.K.H.); 6 Division of Clinical Pharmacology, Vanderbilt University Medical Center, Nashville, TN 37232, USA; maciej.buchowski@vumc.org; 7 Division of Pediatric Neurology, Department of Pediatrics, Vanderbilt University Medical Center, Nashville, TN 37232, USA; william.b.burnette@vumc.org; 8 Department of Biology, Western Kentucky University, Bowling Green, KY 42101, USA

**Keywords:** brain-derived neurotrophic growth factor, dilated cardiomyopathy, rs6365 polymorphism, Duchenne muscular dystrophy, Val66Met

## Abstract

Brain-derived neurotrophic factor (BDNF) is a neuronal growth and survival factor that harbors cardioprotective qualities that may attenuate dilated cardiomyopathy. In ~30% of the population, BDNF has a common, nonsynonymous single nucleotide polymorphism rs6265 (Val66Met), which might be correlated with increased risk of cardiovascular events. We previously showed that BDNF correlates with better cardiac function in Duchenne muscular dystrophy (DMD) patients. However, the effect of the Val66Met polymorphism on cardiac function has not been determined. The goal of the current study was to determine the effects of rs6265 on BDNF biomarker suitability and DMD cardiac functions more generally. We assessed cardiovascular and skeletal muscle function in human DMD patients segregated by polymorphic allele. We also compared echocardiographic, electrophysiologic, and cardiomyocyte contractility in C57/BL-6 wild-type mice with rs6265 polymorphism and in *mdx*/mTR (mDMD) mouse model of DMD. In human DMD patients, plasma BDNF levels had a positive correlation with left ventricular function, opposite to that seen in rs6265 carriers. There was also a substantial decrease in skeletal muscle function in carriers compared to the Val homozygotes. Surprisingly, the opposite was true when cardiac function of DMD carriers and non-carriers were compared. On the other hand, Val66Met wild-type mice had only subtle functional differences at baseline but significantly decreased cardiomyocyte contractility. Our results indicate that the Val66Met polymorphism alters myocyte contractility, conferring worse skeletal muscle function but better cardiac function in DMD patients. Moreover, these results suggest a mechanism for the relative preservation of cardiac tissues compared to skeletal muscle in DMD patients and underscores the complexity of BDNF signaling in response to mechanical workload.

## 1. Introduction

Brain-derived neurotrophic growth factor (BDNF) is an essential mediator of neuronal growth, differentiation, and survival and plasticity [1]. BDNF is also important for development of the cardiac microvasculature, basal cardiac contractility in the postnatal heart, and response to cardiac injury [2]. BDNF is produced as a proprotein (proBDNF), which upon cleavage releases an N-terminal fragment (prodomain) and C terminus that represents mature BDNF (mBDNF) [3]. Processed mBDNF binds to tyrosine kinase receptor B (TrkB), which transduces BDNF’s mitogenic, pro-survival, and cardioprotective benefits. Proteolytic cleavage of proBDNF releases a functional N-terminal prodomain along with BDNF. Once liberated, the BDNF prodomain binds to and regulates trafficking and secretion of mBDNF [3,4].

In approximately 30% of the general population, the prodomain of BDNF has a nonsynonymous (G→A) single nucleotide polymorphism (SNP) at codon position 66 (rs6265), resulting in replacement of valine (Val) with methionine (Met) [5]. The Val66→Met substitution substantially increases the binding stability of the prodomain to mature BDNF, consequently reducing BDNF’s trafficking and secretion [6,7]. Val→Met may additionally alter receptor binding and signaling of proBDNF [8], as well as confer biological activity on the secreted prodomain itself [4]. The rs6265 polymorphism has been implicated in the risk of cardiovascular disease, although results from different clinical studies are conflicting. In one study [9], the Met allele was associated with obesity in patients with coronary artery disease (*n* = 206) but not in otherwise healthy individuals (*n* = 498), whereas the Met allele was apparently protective compared to the Val/Val genotype in a separate cohort of 5510 patients assessed for severity of coronary artery disease and incidence of clinical cardiovascular disease events [10]. The potential contribution of rs6265 to human disease is further complicated by substantive variability in allelic frequency among different populations (e.g., <1% in Sub-Saharan Africans, ~7–35% in Europeans, and ~44% in Asians) [5,11,12].

We previously demonstrated an association of circulating BDNF with better heart function in a small cohort of Duchenne muscular dystrophy (DMD) patients [13]. However, when we added additional patients to our analysis, this positive relationship between BDNF levels and left ventricular ejection fraction (LVEF) was no longer significant, prompting us to investigate whether or not the BDNF rs6265 polymorphism might contribute to these extemporaneously negative findings. In this study, we tested the hypothesis that the Val→Met conversion reduces bioavailability of functional BDNF in DMD patients, thereby leading to reduced function compared to non-carriers. Although we confirmed our original findings that circulating BDNF correlates positively with cardiac function in DMD patients who express normal BDNF [13], here we show that DMD patients who are carriers of the rs6265 allele exhibit better (rather than worse) cardiac function, when compared to age-matched non-carriers. Using a knock-in mouse model (Val66Met mice), which harbors the BDNF G→A substitution, we discovered a role for the Val66Met polymorphism in modulating cardiomyocyte contractility as a possible mechanism contributing to altered heart function in the context of dilated cardiomyopathy. This is the first study implicating the rs6265 polymorphism in DMD or in skeletal muscle function in a progressively destructive neuromuscular disease.

## 2. Results

### 2.1. BDNF rs6265 (Val66Met) in DMD Patients

**Figure 1:**
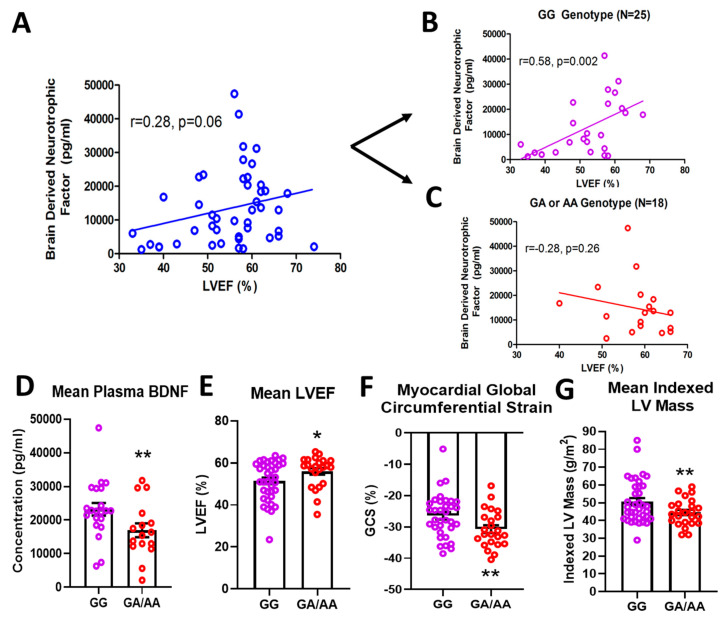
Effects of rs6265 polymorphism on cardiac phenotype. There is a disparate relationship between brain-derived neurotrophic factor (BDNF) levels and left ventricular ejection fraction (LVEF) for Duchenne muscular dystrophy (DMD) patients segregated by rs6265 polymorphism. (**A**) plot for the entire DMD subset cohort (*n* = 43) demonstrating positive correlation of plasma BDNF concentration (pg/mL) vs. LVEF (%) by cardiac magnetic resonance (CMR) (r = 0.28, *p* = 0.06). When segregated by rs6265 polymorphism, a similar positive correlation (r = 0.58, *p* = 0.002) was seen for GG (Val/Val) DMD patients (**B**) but a negative correlation was observed (r = −0.28, *p* = 0.26) for GA (Val/Met) and AA (Met/Met) DMD patients (**C**). GA/AA carriers had lower plasma levels of BDNF (**D**), higher mean LVEF (*n* = 37 vs. 24) (**E**), better global circumferential strain (GCS) (*n* = 37 vs. 24) (**F**), and lower indexed LV mass (*n* = 37 vs. 24) (**G**). Comparisons by two-way ANOVA or Welch’s t-test. Asterisk represents statistical significance for GG versus GA/AA, * *p* < 0.05, ** *p* < 0.03. Individual patients (unsegregated) are indicated by blue circles. Purple circles indicate patients without (GG) the BDNF polymorphism. Rec circles denote those patients with one (GA) or both (AA) BDNF rs6265 alleles.

In a cohort of 61 DMD patients genotyped for the rs6265 allele, 61.7% (37/61) were non-carriers (GG) and 36.7% (23/61) were single allelic carriers (GA). Only one person (1/61 or 1.7%) had the Val66Met conversion for both alleles (AA) (Table 1). In a subset of patients for whom cardiac magnetic resonance (CMR) was performed and circulating BDNF levels measured, there was a modest but insignificant association (*n* = 43, r = 0.28, *p* = 0.06) between BDNF and LVEF (Figure 1A). This positive correlation increased substantially (r = 0.58) and reached significance (*p* = 0.002) for non-carriers only (*n* = 25) (Figure 1B). For carriers (*n* = 18), BDNF levels were negatively correlated with LVEF (r = 0.28) (Figure 1C), but this association was not statistically significant. Overall, carriers had lower mean plasma BDNF levels compared to non-carriers (16,957 ± 2066 pg/mL, *n* = 18 vs. 23,217 ± 1894 pg/mL, *n* = 25, *p* = 0.032, Figure 1D). Carriers also had modestly higher but significant mean LVEF compared to non-carriers (56.0 ± 1.6%, *n* = 23, vs. 51.5 ± 1.5%, *n* = 37, *p* = 0.044, Figure 1E) and better global circumferential strain (−30.7 ± 1.3%, *n* = 23 vs. −26.5 ± 1.2%, *n* = 34, *p* = 0.017, Figure 1F). There was not a significant difference in angiotensin converting enzyme inhibitor (ACEI)/angiotensin receptor blocker (ARB) or beta-blocker use between carriers and non-carriers, although steroid use was more common in carriers (Table 1). Interestingly, carriers had significantly lower indexed left ventricular (LV) mass (44.5 ± 1.5 g/m^2^, *n* = 23, vs. 50.5 ± 2.0 g/m^2^, *n* = 37, *p* = 0.032, Figure 1G) and indexed end-diastolic volumes (61.0 ± 2.5 mL/m^2^, *n* = 23, vs. 71.1 ± 3.7 mL/m^2^, *n* = 37, *p* = 0.05).

**Figure 2:**
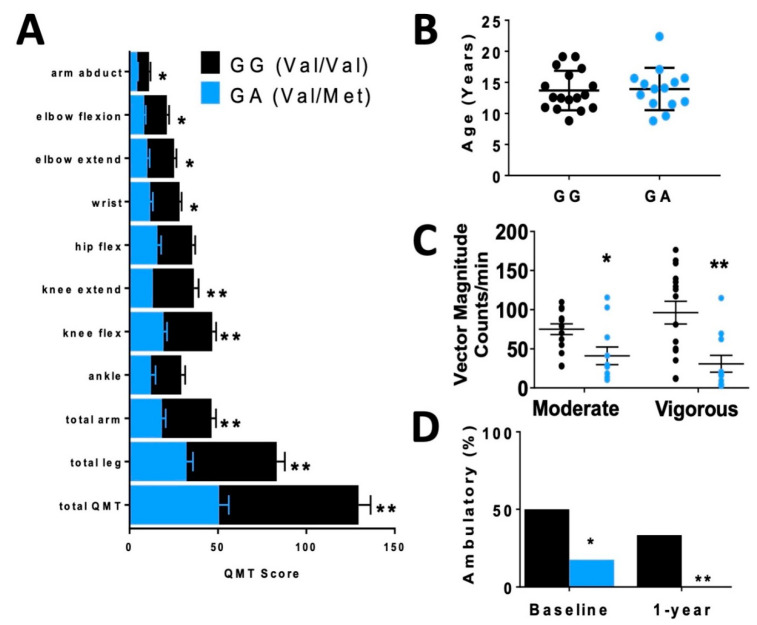
Disparate skeletal muscle performance in DMD patients when compared by rs6265 polymorphism. (**A**) Comparative bar graphs of quantitative muscle testing (QMT) scores for several muscle groups compared by rs6265 phenotype for GG (Val/Val) and GA (Val/Met). All muscle groups in the Val/Met patients showed significantly lower scores. (**B**) There was no difference in mean age between the phenotype groups. (**C**) Accelerometry data demonstrated Val/Met patients had significantly lower performance with moderate and vigorous exercise compared to Val/Val patients (41 ± 11 counts/min, *n* = 11, vs. 75 ± 7 counts/min, *n* = 15, * *p* = 0.031, and 31 ± 11, *n* = 11, vs. 96 ± 15, *n* = 15, ** *p* = 0.003, respectively). Comparisons by two-way ANOVA. (**D**) Age-similar Val/Met DMD patients were less likely to be ambulatory at baseline assessment and at 1 year follow-up.

In order to assess differences in functional status between allelic groups, we assessed skeletal muscle strength and activity level. Skeletal muscle function was more significantly impaired in carriers, based on quantitative muscle testing (QMT) (Figure 2A), accelerometer (Figure 2C) and percentage of patients who were ambulatory (Figure 2D) relative to non-carriers of similar age (Figure 2B).

### 2.2. Cardiac Characterization of Val66Met Mice

**Figure 3:**
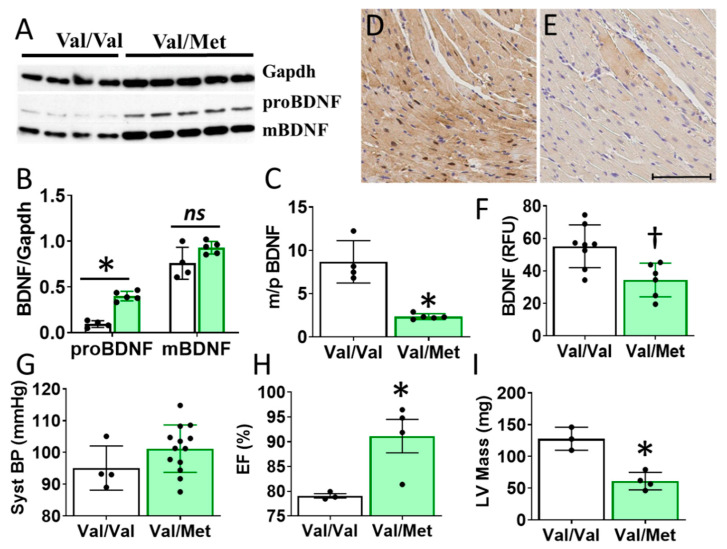
Val66Met mice have increased BDNF in tissue lysates and altered cardiac function as measured by echocardiography. (**A**) Western blot of left ventricular cardiac tissues from Val66Met mouse for proprotein brain-derived neurotrophic factor (proBDNF) (~27 kDa, middle) and mature BDNF (mBDNF) cleavage product (~14 kDa, bottom). (**B**) Bar graph of densitometry analysis of pBDNF and mBDNF normalized to gapdh (y axis) for Val/Val (white bars, *n* = 4) and Val/Met (green bars) (*n* = 5). (**C**) Bar graph showing processed BDNF as a ratio of mBDNF/proBDNF (m/p). (**D,E**) Anti-BDNF-DAB stained hearts from Val/Val (**D**) and Val/Met (**E**) mice at 20X magnification, scale bar = 100 μm. (**F**) Bar graph showing quantification of whole hearts from V (*n* = 8) and Val/Met (*n* = 6) mice relative to total tissue area. (**G**) Plot showing systolic blood pressure (mmHg) as measured by tail cuff for Val/Val (*n* = 4) and Val/Met (*n* = 13). (**H,I**) Results of echocardiography assessment of Val/Val (white bars, *n* = 3) and Val/Met (green bars, *n* = 4) mice are shown. Parameters shown (y axis) are (**H**) percent (%), ejection fraction (EF); (**I**) LV mass (corrected). Asterisks represent statistical significance for V versus VM * *p* < 0.0001 (**B**), * *p* < 0.0006 (**C**), † *p* = 0.0079 (**F**), * *p* = 0.0302 (**H**), * *p* = 0.0026 (**I**).

Cardiac BDNF (27kDa) was significantly higher in whole heart lysates of mice with the BDNF rs6265 (Val/Met, VM) polymorphic allele compared to non-carrier (Val/Val, V) littermate controls (Figure 3A,B). There was no significant difference in cleaved BDNF (14 kDa) between VM and V (Figure 3C). Although this finding is counter intuitive to lower circulating blood plasma levels in DMD patients (Figure 1D), the results are consistent with the reported role of the rs6265 SNP in processing and trafficking of mature BDNF in neuronal cell cultures [7]. There could be lower bioavailability of BDNF, which is suggested by a lower ratio of mature BDNF (mBDNF) to unprocessed proBDNF (Figure 3C) and histological assessment of whole heart slices (Figure 3D). 

Consistent with what was observed in human DMD patients, Val/Met mice exhibited increased left ventricular (LV) ejection fraction (EF, Figure 3C) and lower LV mass (Figure 3D), as measured by echocardiography. Asterisks indicate * *p* < 0.01 or ** *p* < 0.001.

### 2.3. Cardiomyocyte Contractility in Val66Met Mice

**Figure 4:**
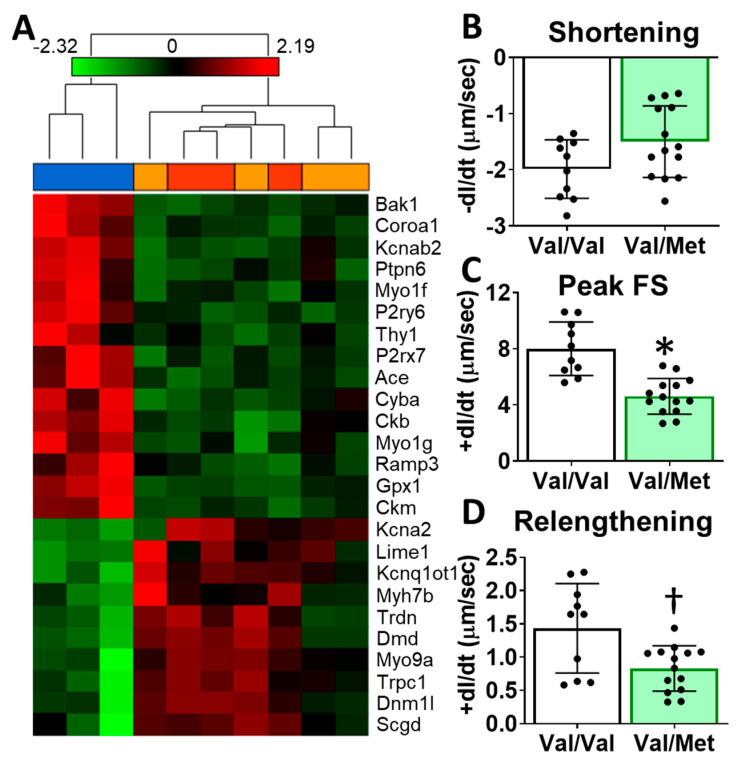
Val66Met mice have altered cardiac tissue gene expression of contractile proteins and reduced cardiomyocyte contractility. (**A**) Hierarchical cluster of cardiac-associated genes identified as significantly differential (FDR-corrected *p* < 0.01, fold-difference > 1.5) using deep sequencing. Vertical dendrograms and columns represent individual samples from whole hearts of Val/Val (blue), Val/Met (orange), and Met/Met (red) mice. Rows represent individual transcripts, indicated by official gene symbol. Heat map colors represent the highest (bright red), lowest (bright green) and median (black) expression levels. (**B**–**D**), Contractility measurements of isolated cardiomyocytes from Val/Val (V, white bars, *n* = 10) and Val/Met (VM, green bars, *n* = 14) mice showing the change in length (dl) over time (dt). Comparisons by two-way ANOVA. Asterisk represents statistical significance for V versus VM * *p* < 0.0001, † *p* = 0.0084.

To identify possible mechanisms that could account for altered cardiac functions in mice with the human BDNF rs6265 polymorphism, we performed transcriptome sequencing of whole hearts from Val66Met mice. Differentially expressed genes included myosin heavy chains, ion channels, and calcium regulators. Gene enrichment analysis revealed two notable cardiac-specific functions: heart contraction (*p* = 0.0148, see Supplementary Table S1 for additional categories) and regulation of calcium ion transport into the cytosol (*p* = 2.45 × 10^−4^) (Figure 4A). Consistent with sequencing results, we noted differences, although insignificant, in cardiomyocyte sarcomere shortening between Val/Met and Val/Val genotypes (Figure 4B). Peak fractional shortening and re-lengthening were significantly reduced in Val/Met cardiomyocytes, compared to those from Val/Val littermate controls (Figure 4C,D).

### 2.4. Val66Met in a Model of Dilated Cardiomyopathy

**Figure 5:**
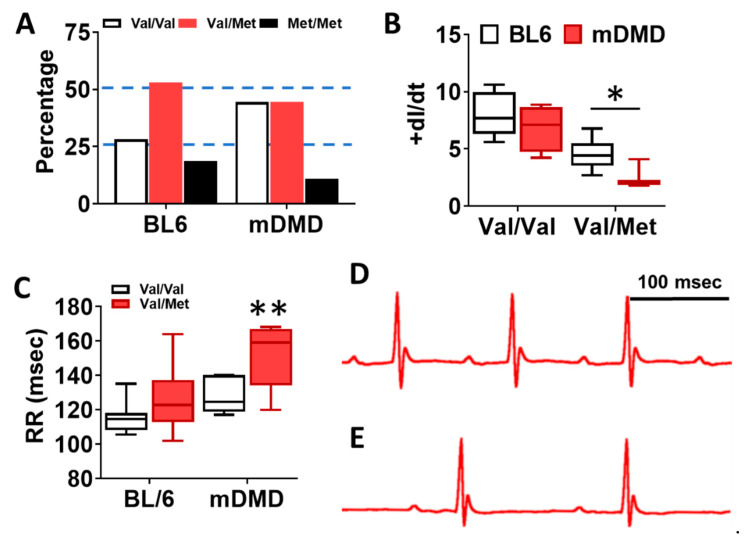
Dystrophic mice have altered distribution of rs6265 polymorphisms, altered cardiomyocyte contractility, and bradycardia. (**A**) Plot showing genotypic distribution of offspring from Val/Met breeding pairs on a normal C57BL/6 (BL6) or mdx/mTR dystrophic (mDMD) background. Percentage of offspring for each of the three possible genotypes are shown on the y axis, with the expected Mendelian ratio of 1:2:1 (or 25%, 50%, 25%) indicated by dashed lines. Val/Val (white), Val/Met (red), and Met/Met (black). (**B**) Box and whisker plot showing peak fractional shortening as the change in the length over time (+dl/dt) of cardiomyocytes isolated from Val/Val or Val/Met mice on a BL6 (*n* = 10, *n* = 14) or mDMD (*n* = 4, *n* = 3) background. (**C**) Plot showing the RR interval as measured by electrocardiography of Val/Val (white) and Val/Met (red) mice on the C57BL/6 (BL6, *n* = 8, 18) or mdx/mTR (mDMD, *n* = 6, 3) background. Example electrocardiograms are also shown for Val/Val (**D**) and Val/Met (**E**) mice on the mDMD background. * Asterisk represents statistical significance (* *p* = 0.0282, Student’s *t*-test, *p* = 0.0032, two-way ANOVA), ** *p* = 0.0025 (two-way ANOVA).

**Figure 6:**
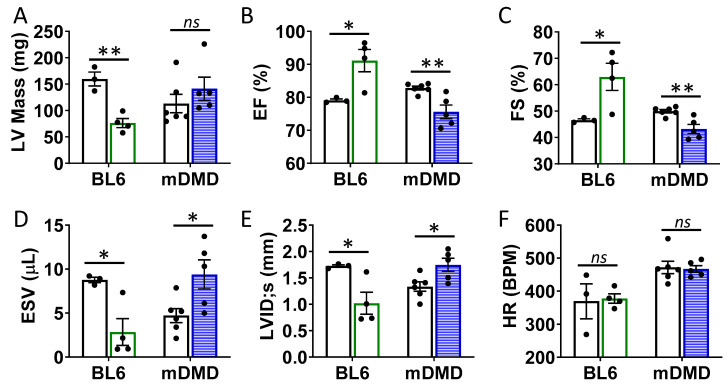
Val66Met leads to decreased ventricular function and increased end diastolic volumes in dystrophic mice. (**A**) Plot showing results of echocardiography assessment of mice with the rs6265 Val/Met polymorphism on a normal C57BL/6 (BL6, green bars) or mdx/mTR dystrophic (mDMD, blue bars) background relative to non-carrier controls (white bars). Parameters shown (y axis) are (**A**) left ventricular (LV) mass, (**B**) percent (%) ejection fraction (EF), (**C**) fractional shortening (FS), (**D**) end systolic volume (ESV), (**E**) left ventricular internal dimension at end systole (LVID;s) and (**F**) heart rate (HR). *n* = 4 (BL6), *n* = 5 (mDMD). Asterisks represent statistical significance. * *p* < 0.05, ** *p* < 0.01, ns = not significant.

To assess the effects of the Val66Met allele in a model of progressive cardiac contractile dysfunction and dilated cardiomyopathy and Duchenne muscular dystrophy (DMD), we crossed Val66Met mice with mdx/mTR (mDMD) mice, which develop cardiac fibrosis and dilated cardiomyopathy that is pathophysiologically comparable to the human disease. Observationally, Val66Met mice on the C57BL/6J background produce offspring with a phenotypic Mendelian ratio of 1:2:1 as expected. However, homozygous carriers of the rs6265 allele (Met/Met mice) were rarely observed on the mDMD background due in part to death shortly after birth (Figure 5A). Cardiomyocyte contraction (peak fractional shortening (FS)) was also significantly reduced in mDMD Val/Met mice compared with Val/Met on the C57BL/6 background (Figure 5B). Cardiac electrophysiology was significantly altered in Val/Met on the mDMD background (Figure 5C,D), with significantly elongated RR interval in mDMD mice with the rs6265 allele, compared to littermate controls (Figure 5E). In contrast to what was observed on the BL6 background, the rs6265 allele reduced cardiac function, as demonstrated by increased systolic diameter and volume and reduced ejection fraction and fractional shortening in mDMD; Val/Met mice (Figure 6).

### 2.5. Acute Cardiovascular Functions in Response to BDNF Receptor Inhibition

**Figure 7:**
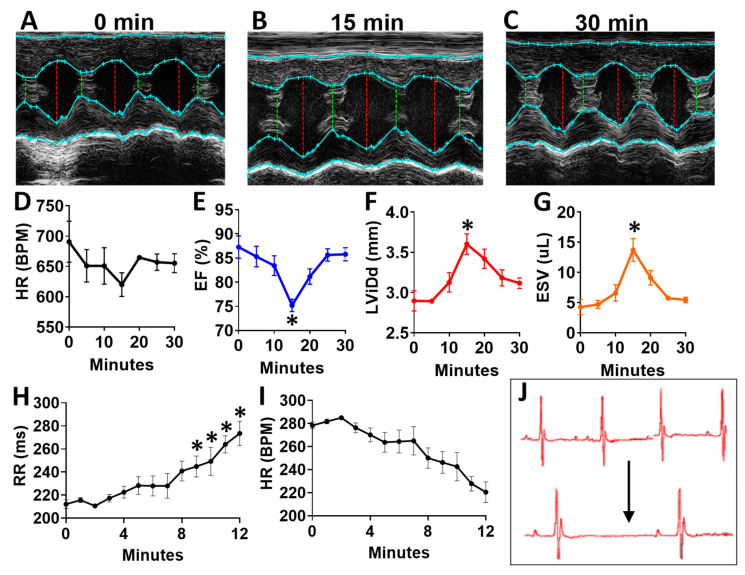
N-[2-[[(Hexahydro-2-oxo-1H-azepin-3-yl)amino]carbonyl]phenyl]benzo[b]thiophene-2-carboxamide (ANA12) injection leads to acutely altered ventricular function and bradycardia in wild-type animals (**A**) Example M-mode echocardiographic traces at baseline (**A**) and at 15 min (**B**) and 30 min (**C**) after a single injection of ANA-12. Plot showing results of echocardiography assessment in mice (*n* = 4) at baseline and at 5 min intervals, including the following echocardiographic parameters (y axis) (**D**) heart rate, (**E**) percent (%) ejection fraction (EF) and (**F**) end systolic volume (ESV) and left ventricular internal dimension at **G,** diastole (LVID;d); *n* = 4. Asterisks represent statistical significance. For EF (* *p* = 0.0183, *n* = 4), LVID; d (* *p* = 0.0169, *n* = 4), and ESV (* *p* = 0.0190, *n* = 4). Plots showing the RR interval (**H**) and heart rate (HR, **I**) as measured by electrocardiography in ANA-12 injected wild-type mice (*n* = 4). Example electrocardiograms are also shown (**J**) at baseline (top) and at 12 min after ANA-12 treatment (bottom). Asterisks represents statistical significance (* *p* < 0.01, *n* = 4). Colors are for emphasis as follows: blue = EF%, red = LViDd, orange = ESV.

To determine the acute effects of systemic inhibition of BDNF signaling, we intraperitoneally injected wild-type (WT) mice with 500 ng/kg N-[2-[[(Hexahydro-2-oxo-1H-azepin-3-yl)amino]carbonyl]phenyl]benzo[b]thiophene-2-carboxamide (ANA-12) [14,15], a specific, small molecule inhibitor of the BDNF tyrosine kinase receptor (TrkB), and monitored mice by echocardiography at baseline (Figure 7A) and every five minutes for 30 total minutes. ANA-12 acutely reduced cardiac function, with a peak response at 15 min (Figure 7B) and essentially normalized function by 30 min (Figure 7C), as demonstrated by a slight decrease in heart rate (Figure 7D), decreased left ventricular (LV) ejection fraction (Figure 7E), increased diastolic diameter (Figure 7F) and end-systolic volume (Figure 7G) in WT mice. Cardiac electrophysiology was also significantly altered in response to ANA-12 injection (Figure 7H–J), with significantly elongated RR interval (Figure 7H) within three minutes that lasted for the duration of monitoring time (~12 min). This was accompanied by a slight decrease in heart rate (Figure 7I) with clear bradycardia as shown in representative electrocardiography traces (Figure 7J).

## 3. Discussion

There is controversy over the importance of circulating BDNF and the utility in correlating with cardiovascular function [10,16,17,18]. Our data provide strong evidence that circulating BDNF levels are correlative with functional markers when segregated by the rs6265 polymorphism. The Val66→Met polymorphism is human-specific [19]. To study the molecular mechanisms underlying differences in cardiac function, Dr. Francis Lee generated the polymorphism in mice [20]. These mice are more prone to anxiety [20] and have been used extensively to study a variety of neurological and psychiatric disorders [21]. However, the cardiac phenotype of these mice has not been examined. In Val66Met mice, we observed significant changes in LV function with a decrease in LV mass that mirrored findings in humans. One major discrepancy, however, was increased BDNF in murine tissues (Figure 2A,B) but decreased BDNF levels in blood plasma of humans with DMD (Figure 1E). Unfortunately, these disparate findings cannot be easily addressed experimentally, as circulating BDNF is essentially undetectable in mice, larger Val66Met animals in which plasma BDNF can be measured have yet to be produced and obtaining biopsies from genotyped patients for measuring BDNF tissue levels is highly problematic, especially in the DMD population.

Despite having higher cardiac levels of BDNF and an increase in LVEF at baseline, Val66Met mice had abnormal cardiomyocyte contractility, which is consistent with gene expression alterations in contractile and calcium handing domains. Thus, this polymorphism appears to predispose cardiac muscle to abnormal contraction, and while there may be compensatory mechanisms under basal conditions, there may be less contractile reserve and a higher risk of developing functional decline under pathological conditions. This finding was consistent with CMR data from DMD patients demonstrating decreased LV mass but increased LVEF and better GCS. While this may at first appear counterintuitive, recent evidence in young adult cancer survivors showed heart failure symptoms correlated with decline in LV mass and not necessarily with LVEF [22]. In DMD patients, the lower LV mass and worse skeletal muscle function may represent higher myocyte turnover leading to ultimately faster disease progression. It should also be noted that significantly more carriers were on steroids (89% vs. 60%), which is most likely due to the poorer skeletal muscle function. It is known that steroids have cardioprotective effects [23,24]; therefore, some of the improved parameters of LV function could be secondary to earlier and more consistent steroid use and may delay the decreased function seen in the murine studies. 

Given the correlation of BDNF levels with cardiovascular risk in the general population and the fact that BDNF is produced by and acts upon cardiomyocytes to directly impact contractility [25], it is possible that lower expression of BDNF in the heart is associated more generally with dilated cardiomyopathy (DCM). A meta-analysis of the Gene Expression Omnibus (GEO) [26,27] repository gene expression data of cardiomyocytes derived from human induced pluripotent stem cells (GSE13834, GSE35108) and cardiac tissues of various etiologies of end-stage human heart failure, including DCM (GSE42955) [28] supports this contention (Supplementary Figure S1A). Further, the gene encoding the receptor for BDNF is significantly up-regulated (*n* = 27, *p* = 0.002) in explants from patients with end-stage human heart failure, compared to unused donor healthy heart controls (*n* = 16) (Supplementary Figure S1B), perhaps as a compensatory response to reduced BDNF levels. To determine the effect of the rs6265 polymorphic alleles in such a disease state, we chose to use the mDMD mouse as a model for progressive dilated cardiomyopathy DCM. This highlights an important possible mechanism for BDNF effects in DMD specifically and DCM more generally. TrkB-T1 receptors have been demonstrated to be critical for normal contractility in both skeletal [29] and cardiac [25] myocytes. Selective cardiac deletion of TrkB-T1 results in decreased contractile force generation and the development of cardiomyopathy [25]. Altered active BDNF excretion or altered BDNF-induced receptor activation are possible mechanisms that will need further exploration in future studies. It will also be important to assess the effects of the rs6265 polymorphism over an extended time frame to assess disease progression and determine whether disparate results are due to compensatory effects that in older mice would be progressive and more closely resemble what is seen in humans. 

DMD cardiomyopathy is characterized by muscle fibrosis, loss of contractility, and progressive development of DCM. The apparent disparate correlations between plasma BDNF levels and LVEF observed when segregating by polymorphic allele in DMD patients may help to partially explain the controversial effects of BDNF in human disease. Another universal feature of DMD muscle cells is that the absence of dystrophin leads to altered calcium handling through multiple mechanisms [30]. This has also been demonstrated in dystrophic murine skeletal and cardiac muscle cells [31], providing the opportunity to ascertain whether reduced contractility was due to altered calcium handling alone or other contributing mechanisms. Expression of the polymorphic alleles in the murine mDMD model led to substantial functional differences in addition to heart rate reduction, including decreased LVEF and FS, as well as increased end diastolic volumes. If this effect were primarily due to alteration in calcium handling, such a profound difference between Val/Val mDMD cardiomyocytes and the Val/Met and Met/Met groups would not be expected, as all of the groups would have abnormal calcium regulation. This suggests that altered BDNF metabolism and trafficking and/or altered regulation of contractility genes plays a significant role in the phenotype and may represent a novel role for this polymorphism as a modulator of cardiovascular disease. We suspect that the seemingly contradictory findings in LV function on DMD patients and in mDMD animals may represent a period of relative compensation as cardiomyocyte turnover increases while abnormally contractile cells undergo apoptosis. We would predict that as DMD boys are followed over time we would see LVEF ultimately segregate by rs6265 polymorphism. 

The striking difference seen in skeletal muscle performance in DMD patients segregated by the Val66Met polymorphism was not entirely surprising, given BDNF’s known role as a mitokine and in promoting exercise-induced skeletal muscle regeneration [32]. As DMD affects both cardiac and skeletal muscle, leading to the abnormal calcium handling and muscle weakness that is characteristic of the disease, it is unclear if rs6265 would have a similar, detectible effect in the general population. It is more likely that this functions in a “two hit” mechanism, where the abnormal contractility of myocytes can be compensated for during normal conditions, however in diseased states, such as DMD or other forms such as DCM, this fragile balance is disrupted leading to a worse clinical phenotype.

For the first time, we have shown that a polymorphism in the BDNF gene leads to functional changes in both mouse models and humans with DCM and in skeletal muscle function in DMD patients. This will allow for further investigation into the mechanisms of altered cardiomyocyte contractility and may provide a novel component of risk assessment in patients with cardiomyopathy or other cardiovascular diseases.

## 4. Materials and Methods

### 4.1. Animals

This study was carried out in accordance with the National Institutes of Health’s Public Health Service Policy of Humane Care and Use of Laboratory Animals and the Animal Welfare Act. Transgenic BDNF_Met_ knock-in allele (Val66Met) mice [20] were crossed with first generation mdx/mTR KO (B6.Cg-*Terc^tm1Rdp^*/*Dmd^mdx−4Cv^*/BlauJ, Jackson #023535) [33], which develop progressive cardiac fibrosis and dilated cardiomyopathy [34]. Litter mates (8–12 weeks old) were used wherever possible. For all experiments, animals were euthanized with either isoflurane followed by tissue isolation or via carbon dioxide asphyxiation. For treatments with the TrkB receptor antagonist N-[2-[[(Hexahydro-2-oxo-1H-azepin-3-yl)amino]carbonyl]phenyl]benzo[b]thiophene-2-carboxamide (ANA-12) (Tocris Bioscience, Minneapolis, MN, USA), mice were injected with a conservative dose (500 ng/kg), diluted in saline solution made from a 1mg/mL stock solution in DMSO (Sigma-Aldrich, St. Louis, MO, USA [14,15]. 

### 4.2. Echocardiography

Transthoracic M-mode echocardiography was performed with a 12-mHz probe (VisualSonics, Toronto, ON, Canada) on conscious mice and on mice anesthetized by inhalation of isoflurane (1–1.5%). LV end-systolic interior dimension (LVID;s), LV end diastolic interior dimension (LVID;d), ejection fraction (EF) and fractional shortening (FS) values were obtained by analyzing data using the Vevo 2100 program (VisualSonics, Toronto, ON, Canada).

### 4.3. Electrocardiography

ECG leads were recorded with surface electrodes (ADInstruments, Colorado Springs CO, USA) on anesthetized mice by constant inhalation of isoflurane (1–1.5%). The mean value for each mouse was obtained from four values consisting of four consecutive cardiac cycles using LabChart software (ADInstruments, Colorado Springs CO, USA). 

### 4.4. Cardiomyocyte Contractility

Adult mouse cardiomyocytes were isolated as described previously [35]. For recordings, myocytes were paced at 1 Hz with a MyoPacer field stimulator (IonOptix, Westwood, MA, USA). Contractility measurements were made using sarcomere length (SarcLen) parameters and processed with IonWizard 6.0 software (IonOptix, Westwood, MA, USA).

### 4.5. Patients

The Vanderbilt Institutional Review Board approved this prospective study, and this investigation conforms with the principles outlined in the Declaration of Helsinki (IRB Protocol numbers 120929, 140049, and 161524). Appropriate consents and assents were obtained as part of study enrollment. Sixty-one DMD subjects were enrolled from the Neuromuscular Cardiology Clinic. Inclusion criteria were: (1) diagnosis of DMD with clinical phenotype and confirmation with either genetic testing or muscle biopsy, (2) blood obtained at time of cardiac MRI (CMR), (3) able to tolerate CMR without sedation or anesthesia. Exclusion criteria were: (1) additional cardiac diagnoses that could affect biomarkers, (2) inadequate volume of blood. Enrolled DMD subjects underwent blood draw, CMR, skeletal muscle strength assessment, and assessment of physical activity levels using accelerometry. 

### 4.6. ELISA

BDNF was measured using the Quantikine BDNF enzyme linked immunosorbent assay (ELISA) kit (R&D systems, Minneapolis, MN, USA, cat no. DBD00), as previously described [13]. 

### 4.7. Cardiac Magnetic Resonance

CMR was performed using a 1.5 Tesla Siemens Avanto (Siemens Healthcare Sector, Erlangen, Germany) and calculations performed as previously described [36]. A peripheral intravenous line was used to administer Gd-DTPA contrast (gadopentate dimeglumine, Magnevist^®^, Bayer Healthcare Pharmaceuticals, Wayne, NJ, USA or gadobutrol, Gadovist^®^, Bayer Healthcare Pharmaceuticals, Wayne, NJ, USA) at a dose of 0.2 mmol/kg. 

### 4.8. Skeletal Muscle Assessment

Quantitative muscle testing (QMT) was performed on DMD subjects using a handheld myometer—an objective, reproducible method for upper and lower extremity strength evaluation in DMD. QMT score was calculated as previously described [37]. QMT was performed in 33 of 35 DMD subjects. Physical activity was assessed using triaxial accelerometers (GT3X+, ActiGraph, Pensacola, FL, USA) that recorded raw accelerometry data at a sampling frequency of 30 Hz (30 observations per second for each axis) for 7 consecutive days and 24 h per day (one monitor on the ankle of the dominant leg and one on the wrist of the dominant hand). Raw accelerometry data were integrated into 15-s epochs and expressed as vector magnitude (VM) counts using Actilife software (ActiGraph, Pensacola, FL, USA). Accelerometer non-wear and wear periods were assessed as previously described [38]. A recording was considered valid if it included ≥3 valid days with ≥2 weekdays and ≥1 weekend night with ≥6 h of monitor wearing from 7:00am to 10:00pm.

### 4.9. Transcriptome Sequencing

RNA sequencing (RNA-seq) was performed by Vanderbilt Technologies for Advanced Genomics (VANTAGE) core as previously described [39], using Illumina TruSeq and HiSeq 3000 (Illumina San Diego, CA, USA)on paired-end-150 flow cell runs at ~32M PF reads per sample. Raw reads (fastq files) were aligned to the mm10 assembly using STAR 2.5.3a and analyzed as previously described using Partek Flow server and Genomics Suite (Partek, Inc, Chesterfield, MO, USA [39]. 

### 4.10. Immunoblotting

Gel electrophoresis was performed as previously described [39] and probed with rabbit anti-BDNF (cat. #ARP41970, Aviva Systems Biology Corporation, San Diego, CA, USA) and rabbit anti-GAPDH (cat. #5174, Cell Signaling Technology, Inc, Danvers, MA, USA).

### 4.11. Statistical Analysis

All data are expressed as means ± SEM. Statistical comparisons made between two variables were performed using the Student’s t test. Comparisons between more than two variables were performed using one-way ANOVA with a Tukey’s post hoc test. Human demographic variables were compared using either a Wilcoxon rank-sum (continuous variables) or a chi-square or Fisher’s exact test (categorical variables). Patient data were collected and managed using research electronic data capture (REDCap) at Vanderbilt [40].

## 5. Limitations

This study is limited by the relatively small population of the study cohort of DMD patients. The number of patients could be potentially increased in future studies using multicenter collaborative data collection. CMR analysis was done separately from BDNF analysis in order to minimize potential bias.

## 6. Translational Perspective

The rs6265 polymorphism alters cardiomyocyte contractility in mice without overt cardiovascular disease and is augmented in a mouse model of progressive dilated cardiomyopathy; thus, it may represent a novel risk factor for worse outcomes in cardiovascular disease. In patients with Duchenne muscular dystrophy, the rs6265 polymorphism is associated with altered relationship between plasma BDNF levels and LVEF and worse skeletal muscle performance.

## Figures and Tables

**Table 1:** Demographics by genotype. Late gadolinium enhancement (LGE) global severity score is ranked from zero (no LGE) to four (extensive involvement of septum and free wall). Group means compared with unpaired t-test for continuous variables or Mann–Whitney rank sum for non-parametric variables. Abbreviations: BSA: Body surface area, LVEF: Left ventricular ejection fraction, LVEDV: Left ventricular end diastolic volume, GCS: Global circumferential strain, GLS: Global longitudinal strain, ACEI: Angiotensin converting enzyme inhibitor, ARB: Angiotensin receptor blocker.

	GG	GA/AA	*p*
	(*n* = 37)	(*n* = 24)	
**Age (years)**	14.9 ± 0.7	14.9 ± 0.9	0.99
**Height (cm)**	150.6 ± 3.1	146.7 ± 3.0	0.39
**Weight (kg)**	54.3 ± 3.5	52.6± 3.5	0.74
**BSA (m^2^)**	1.5 ± 0.1	1.5 ± 0.1	0.67
**LVEF (%)**	51.5 ± 1.5	56.1 ± 1.5	***0.044***
**Heart Rate (bpm)**	94.7 ± 2.8	101.8 ± 3.1	0.099
**Indexed LV Mass (g/m^2^)**	50.6 ± 2.0	44.5 ± 1.5	***0.032***
**Indexed LVEDV (mL/m^2^)**	71.1 ± 3.7	61.0 ± 2.5	***0.05***
**GCS**	−25.3 ± 1.8 (*n* = 34)	−30.7 ± 1.3 (*n* = 23)	***0.03***
**GLS**	−19.6 ± 0.7 (*n* = 30)	−20.4 ± 0.6 (*n* = 20)	0.39
**LGE Global Severity Score**	2.0 ± 0.2	1.7 ± 0.3	0.26
**ACEI or ARB**	32 (87%)	19 (79%)	0.49
**Beta Blocker**	23 (62%)	10 (42%)	0.19
**Steroids**	22 (60%)	21 (89%)	***0.023***

## Supplementary Materials

Supplementary materials can be found at https://www.mdpi.com/1422-0067/21/20/7466/s1.
