# Supplementary material for: The BDNF rs6265 Polymorphism is a Modifier of Cardiomyocyte Contractility and Dilated Cardiomyopathy
Source: Int J Mol Sci. 2020 Oct 10;21(20):7466. doi: 10.3390/ijms21207466 (PMC7593910; doi:10.3390/ijms21207466)
Supplement: Supplementary file 1 [file ijms-21-07466-s001.zip › Supplementary Figure.pptx]

## Slide 1
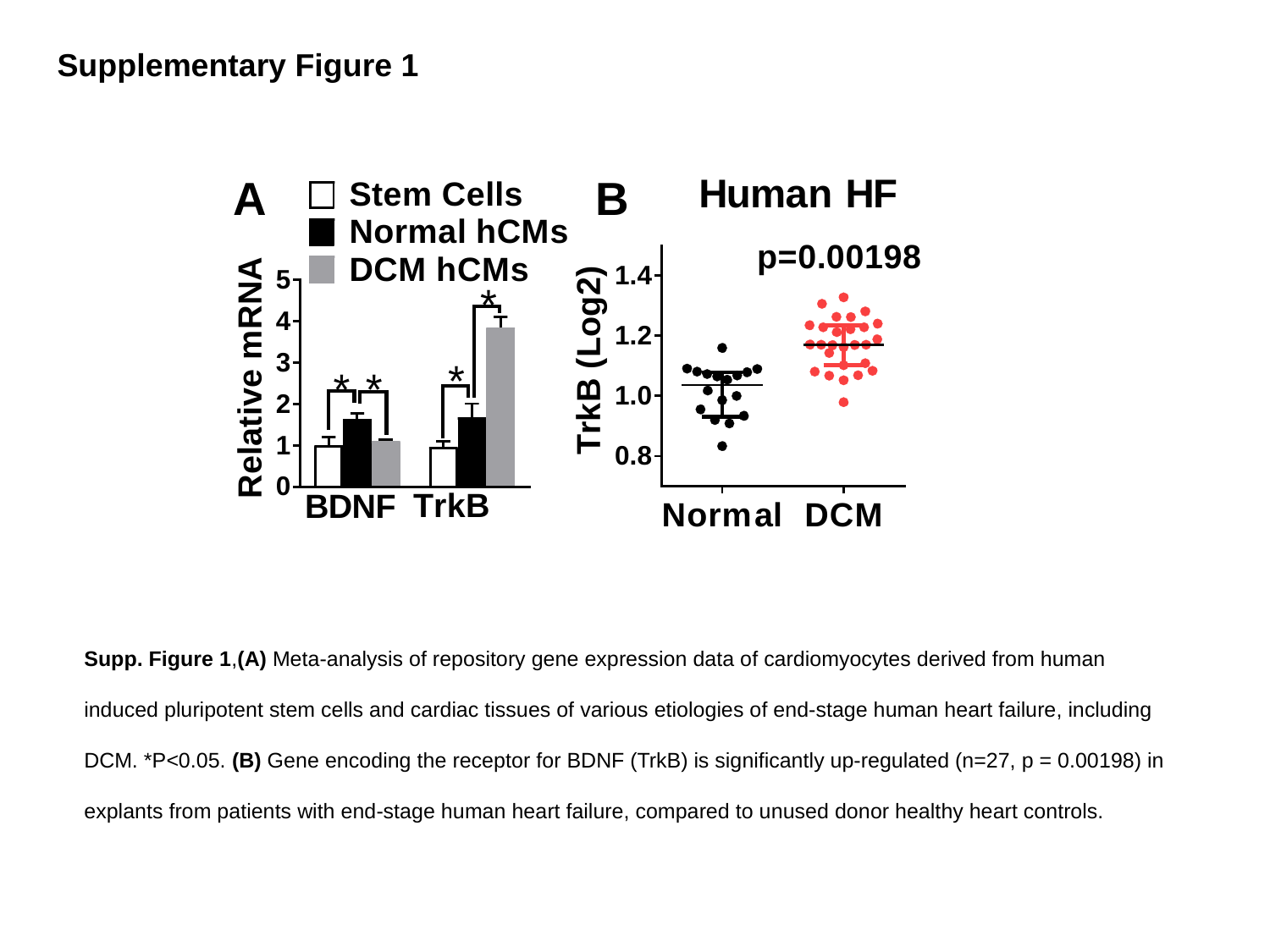

Supplementary Figure 1
Supp. Figure 1,(A) Meta-analysis of repository gene expression data of cardiomyocytes derived from human induced pluripotent stem cells and cardiac tissues of various etiologies of end-stage human heart failure, including DCM. *P<0.05. (B) Gene encoding the receptor for BDNF (TrkB) is significantly up-regulated (n=27, p = 0.00198) in explants from patients with end-stage human heart failure, compared to unused donor healthy heart controls.
