# Supplementary material for: The BDNF rs6265 Polymorphism is a Modifier of Cardiomyocyte Contractility and Dilated Cardiomyopathy
Source: Int J Mol Sci. 2020 Oct 10;21(20):7466. doi: 10.3390/ijms21207466 (PMC7593910; doi:10.3390/ijms21207466)
Supplement: Supplementary file 1 [file ijms-21-07466-s001.zip › Supplementary Table.pdf]

Supplementary Table:

Supplemantary Table 1: Enriched functional categories for genes differentially expressed in cardiac tissues between Val/Val versus Val/Met mice. Columns are as follows: Category = **Gene** Ontology (**GO**) ID; Term = functional description, Count=number of differentially expressed genes in the listed category; % = percent of total genes analyzed included in the category; PValue= p value, Genes=gene symbols of genes in the listed category; FDR= corrected p value

Category	Term	Count	%	PValue	Genes	Fold Enrichment	FDR
GO:0006355	Regulation of transcription, DNA-templated	325	15.6025	1.37E-18	MEF2C, ITGB3BP, MEF2A, ZFP638, ZFP738, BBX, NAA15, RORA, HMGN5, CITED4, EPC2, ZFP933, ZFP932, ZFP931, ZFP930, MED29, CEP290, DHX36, ZFP938, CCAR1, ZFP329, ZFP626, ZFP326, ZHX1, MED13, ZFP74, ZFP81, UHRF1, ASCC3, ZFP280C, ZFP280D, ZZZ3, MAPK8, MAD2L2, MCTS2, ZFP825, TRAPPC2, GM14288, SRSF10, ERBB4, ZFP612, 2610021A01RIK, ZFP758, ZFP458, ZFP759, ZFP617, ZFP953, 2610008E11RIK, ZFP950, DMD, HTATSF1, PBRM1, ZFP959, ZFP958, TCF4, PLAG1, IKZF5, AEBP2, IKZF2, ZFP605, TGFBR1, ZFP97, ZBTB41, ZFP606, ZFP445, DDX5, GM14296, ZBTB44, GCFC2, ABCG1, GM14295, RNF6, ZFP942, SRSF5, ZFP729B, ZFP442, ZFHX4, ZFP944, ZFP943, DMTF1, ZFP946, RNF2, TRPS1, ZFP945, ZFP948, GM4631, ZFP800, KLF2, ZFP729A, CREBRF, NKAP, ENY2, ZFP971, CCNT2, ZFP970, GTF3A, ZBTB33, ZFP433, ZFP40, SPI1, ZEB2, RLIM, ZFP871, ZFP873, MBTD1, GM14391, HLX, ZFP979, ZFP976, GM14399, USP16, THRSP, MYC, SCX, ZFP975, ZFP973, ZFP960, ZBTB20, SNAPC2, SNAPC1, SNAPC3, SCAI, AW146154, CCNC, ZFP37, CCND1, TAF13, ZFP780B, PFDN5, ASH1L, AU041133, ZFP966, RBM39, ZFP968,	1.575204	2.58E-15

					ZFP229, CLOCK, ZFP420, ZFP62, THAP7, 2610044O15RIK8, ZFP65, LCORL, ZFP715, GM10778, ZFP992, ZBTB6, ZBTB9, ZFP991, BDP1, ZFP712, ZFP68, TXLNG, ZBTB17, ZBTB18, 2010315B03RIK, GM13212, CIR1, NR1D2, RB1CC1, CSDE1, ZSCAN26, ETV1, TRP53INP1, ZFP994, PRKAA2, USP34, ETV5, MYSM1, ZFP72, ZFP52, ZFP51, ZFP982, ZFP119B, ZFP119A, PHF10, SNAI2, STAT1, HNRNPDL, ZFP709, ZFP101, IWS1, ATRX, ZFP846, ZFP984, ZFP60, ZBTB1, MPHOSPH8, CBX3, ZXDB, ZFP788, CNOT6, PNN, ESF1, CASP8AP2, MIER1, TARDBP, ZFP131, MIER3, PSIP1, ZFP689, GABPA, ZFP595, MTA1, ZFP597, JUNB, CARF, SLTM, SUZ12, ZFP120, TRIM33, MGA, ZFP260, AKAP8, LITAF, ZFP763, ATF1, GM14440, DDX3X, CNOT6L, ZFP157, ZFP760, HNRNPD, CREBZF, MAFB, TAF7, ZMYM5, ATAD2, BRCA2, ZFP148, UIMC1, FOXP2, SAFB2, FAM208A, GM14327, GM14326, ZFP141, GM14325, HDAC2, ZFP770, CDKN2AIP, RFX3, HDAC9, GM6710, BMI1, E2F2, AEBP1, GPBP1, ARID4B, PRRX1, ZKSCAN3, TMF1, FUBP1, PCGF5, GTF2A1, INO80D, KMT5B, ZFP518A, GM14308, KDM7A, LRIF1, ZFX, CCNL1, CDK8, PKN2, RUNX1T1, NCOA7, ZFP322A, HMGA1, DAPK3, ZFP26, PURA, CCNL2, PRKCB, ZFP654, ZFP160, ITGB1BP1, JMJD1C, LCOR, GM14305, COMMD5, ZFP367, C1D, BCLAF1, NDN,		

					PAXBP1, NR3C1, AI987944, CCDC59, GM14403, CHD9, CHD2, CEBPZ, NFAT5, CHD1, ZFP292, PHF20L1, CEBPA, GM3055, CEBPD, ZFP386, CEBPG, ZFP955B, CREB1, ZFP955A, GM14410, DACH1, SUPT4A, SIRT1, IRF9, ATXN3, ZFP287, IRF5, ZFP182, IRF7, DR1, SP3, ATXN7, IRF8, SP4, PSPC1, ZFP281, ZFP189, SETD2, NFIB, ZBTB8A		

GO:0032776	DNA methylation on cytosine	21	1.008161	9.43E-14	HIST1H4N, HIST2H3B, HIST4H4, HIST2H3C2, HIST2H4, UHRF1, HIST1H4A, HIST1H4B, HIST1H3A, HIST1H3B, HIST1H4F, HIST1H3C, HIST1H3D, HIST1H4C, HIST1H4D, HIST1H3E, HIST1H3F, HIST1H4I, HIST1H3H, HIST1H4H, HIST1H3I	7.248816	1.77E-10
GO:0006335	DNA replication-dependent nucleosome assembly	21	1.008161	2.19E-13	HIST1H4N, HIST2H3B, HIST4H4, NASP, HIST2H3C2, HIST2H4, HIST1H4A, HIST1H4B, HIST1H3A, HIST1H3B, HIST1H3C, HIST1H4F, HIST1H3D, HIST1H4C, HIST1H4D, HIST1H3E, HIST1H3F, HIST1H4I, HIST1H3H, HIST1H4H, HIST1H3I	7.029155	4.12E-10
GO:0045815	Positive regulation of gene expression, epigenetic	21	1.008161	2.19E-13	HIST1H4N, HIST2H3B, HIST4H4, CBX3, HIST2H3C2, HIST2H4, HIST1H4A, HIST1H4B, HIST1H3A, HIST1H3B, HIST1H3C, HIST1H4F, HIST1H3D, HIST1H4C, HIST1H4D, HIST1H3E, HIST1H3F, HIST1H4I, HIST1H3H, HIST1H4H, HIST1H3I	7.029155	4.12E-10

GO:0006334	Nucleosome assembly	34	1.632261	6.40E-11	HIST1H4N, HIST4H4, RSF1, HIST2H3C2, HIST2H2AA1, HIST1H4A, HIST1H2BK, HIST1H4B, HIST1H4F, HIST1H4C, HIST1H4D, HIST1H4I, HIST1H4H, HIST1H2BR, HIST1H2BB, HIST2H3B, HIST1H1D, SHPRH, HIST1H1C, HIST1H1B, HIST1H2BG, MCM2, HIST2H4, ATRX, HIST2H2BB, HIST1H3A, HIST1H3B, SMARCA5, HIST1H3C, HIST1H3D, HIST1H3E, HIST1H3F, HIST1H3H, HIST1H3I	3.611132	1.20E-07
GO:0051290	Protein heterotetramerizatio n	22	1.056169	1.35E-10	HIST1H4N, HIST2H3B, HIST4H4, S100A10, HIST2H3C2, NRXN1, HIST2H4, HIST1H4A, HIST1H4B, HIST1H3A, HIST1H3B, HIST1H4F, HIST1H3C, HIST1H3D, HIST1H4C, HIST1H4D, HIST1H3E, HIST1H3F, HIST1H4I, HIST1H3H, HIST1H4H, HIST1H3I	5.170382	2.54E-07
GO:0000183	Chromatin silencing at rdna	23	1.104177	6.67E-10	HIST1H4N, HIST2H3B, HIST4H4, HIST2H3C2, SIRT1, HIST2H4, HDAC2, HIST1H4A, HIST1H4B, HIST1H3A, SMARCA5, HIST1H3B, HIST1H3C, HIST1H4F, HIST1H3D, HIST1H4C, HIST1H3E, HIST1H4D, HIST1H3F, HIST1H4I, HIST1H3H, HIST1H4H, HIST1H3I	4.619159	1.26E-06

GO:0045653	Negative regulation of megakaryocyte differentiation	13	0.6241	1.16E-09	HIST1H4N, HIST4H4, HIST1H4A, HIST1H4B, GABPA, HIST1H4F, HIST1H4C, PF4, HIST1H4D, HIST1H4I, HIST1H4H, HIST2H4, CIB1	8.4468	2.19E-06
GO:0008380	RNA splicing	51	2.448392	1.92E-08	SRSF1, PRPF4B, LSM8, ZMAT5, ZFP638, RBM5, SYNCRIP, SNRPD2, NSRP1, WBP4, PNN, TARDBP, PTBP3, PTBP2, LUC7L3, ZFP326, FMR1, HNRNPA2B1, PRPF39, MBNL2, MBNL1, HNRNPU, QK, SNRNP48, RBM39, THOC2, PRPF38B, PPP4R2, SRSF10, TRA2A, HNRNPA3, HNRNPM, CIR1, RBM25, PPWD1, PRPF40A, TXNL4B, SREK1, YTHDC1, DDX5, GCFC2, IWS1, SRSF5, SRSF7, CLASRP, RSRC1, ZRANB2, LSM10, HNRNPH1, CWC22, SNRNP25	2.337496	3.62E-05
GO:0060968	Regulation of gene silencing	10	0.480077	2.03E-07	HIST2H3B, HIST1H3A, HIST1H3B, HIST2H3C2, HIST1H3C, HIST1H3D, HIST1H3E, HIST1H3F, HIST1H3H, HIST1H3I	8.496781	3.82E-04

GO:0006397	Mrna processing	59	2.832453	2.76E-07	SRSF1, PRPF4B, AURKAIP1, LSM8, ZMAT5, RBM5, SYNCRIP, SNRPD2, HNRNPLL, NSRP1, WBP4, RNGTT, PNN, TARDBP, PTBP3, PTBP2, PLCB1, LUC7L3, ZFP326, FMR1, HNRNPA2B1, PRPF39, MBNL2, MBNL1, HNRNPU, QK, SNRNP48, CPSF6, RBM39, THOC2, PRPF38B, PPP4R2, SRSF10, TRA2A, HNRNPA3, HNRNPM, CIR1, CNOT6L, NUDT21, RBM27, RBM25, PPWD1, RBM26, PRPF40A, TXNL4B, SREK1, YTHDC1, DDX5, GCFC2, IWS1, SRSF5, SRSF7, CLASRP, RSRC1, ZRANB2, LSM10, HNRNPH1, CWC22, SNRNP25	2.023923	5.20E-04
GO:0006352	DNA-templated transcription, initiation	16	0.768123	2.87E-07	HIST1H4N, HIST4H4, RSF1, TAF7, HIST2H4, HIST1H4A, HIST1H4B, GM4799, SMARCA5, HIST1H4F, HIST1H4C, HIST1H4D, TCF4, HIST1H4I, MYC, HIST1H4H	4.776569	5.40E-04
GO:0006336	DNA replication-independent nucleosome assembly	12	0.576092	4.33E-06	ATRX, HIST1H4N, HIST4H4, HIST1H4A, HIST1H4B, NASP, HIST1H4F, HIST1H4C, HIST1H4D, HIST1H4I, HIST1H4H, HIST2H4	5.301991	0.008149
GO:0019886	Antigen processing and presentation of exogenous peptide antigen via MHC class II	9	0.432069	8.06E-06	FCGR2B, H2-EB1, UNC93B1, IFI30, FCER1G, H2-AA, H2-AB1, H2-DMA, CD74	7.100881	0.015154

GO:0006351	Transcription, DNA-templated	220	10.56169	4.09E-05	MEF2C, ITGB3BP, MEF2A, ZFP638, BBX, NAA15, RORA, HMGN5, CITED4, EPC2, ZFP932, MED29, CEP290, DHX36, CCAR1, ZFP329, ZFP326, ZHX1, MED13, ZFP74, UHRF1, ASCC3, ZFP280C, ZFP280D, MAPK3, ZZZ3, MCTS2, MAD2L2, TRAPPC2, ERBB4, MYEF2, HTATSF1, PBRM1, TCF4, PLAG1, IKZF5, AEBP2, IKZF2, ZFP449, ZBTB41, DDX5, ZFP445, ZBTB44, GCFC2, ZFHX4, SRSF5, DMTF1, RNF2, TRPS1, ZFP800, KLF2, CREBRF, CCNT2, ENY2, NKAP, GTF3A, ZBTB33, SPI1, ZEB2, RLIM, MBTD1, HLX, TWISTNB, USP16, THRSP, MYC, SCX, ZBTB20, SNAPC2, SNAPC1, SNAPC3, SCAI, CCNC, ZFP37, CCND1, TAF13, ASH1L, RBM39, CLOCK, ZFP62, THAP7, LCORL, POLR2L, ZBTB6, ZBTB9, BDP1, TXLNG, ZBTB17, ZBTB18, CIR1, NR1D2, RB1CC1, ZSCAN26, TRP53INP1, ETV1, PRKAA2, MYSM1, PHF10, STAT1, HNRNPDL, SNAI2, IWS1, ATRX, ZBTB1, MPHOSPH8, CBX3, ZXDB, CNOT6, PNN, ESF1, CASP8AP2, MIER1, TARDBP, ZFP131, MIER3, PSIP1, ZFP689, GABPA, MTA1, JUNB, CARF, SUZ12, SLTM, TRIM33, ZFP120, MGA, ZFP260, AKAP8, SWT1, LITAF, ATF1, DDX3X, CNOT6L, HNRNPD, CREBZF, MAFB, ZMYM5, TAF7, ATAD2, ZFP148, UIMC1, SAFB2, FAM208A, FOXP2, PAGR1A, HDAC2, ZFP770, RFX3, HDAC9, BMI1, E2F2, AEBP1, ZKSCAN8, GPBP1, ARID4B, ZKSCAN3, TMF1, FUBP1, PCGF5,	1.289167	0.076832

					GTF2A1, INO80D, KMT5B, ZFP518A, KDM7A, LRIF1, ZFX, CCNL1, PKN2, RUNX1T1, CDK8, NCOA7, ZFP322A, HLTF, DAPK3, CCNL2, PRKCB, PURA, ZFP654, ITGB1BP1, JMJD1C, LCOR, ZFP367, COMMD5, C1D, BCLAF1, NDN, ZFP397, CDC73, NR3C1, PAXBP1, CCDC59, CHD9, NFAT5, CEBPZ, CHD2, CHD1, ZFP292, CEBPA, CEBPD, CEBPG, CREB1, DACH1, SUPT4A, SIRT1, IRF9, ATXN3, ZFP287, IRF5, ZFP182, IRF7, ATXN7, SP3, DR1, SP4, IRF8, PSPC1, ZFP281, SETD2, NFIB, ZBTB8A		
GO:0097421	Liver regeneration	11	0.528084	1.44E-04	SRSF1, SRSF5, CCND1, RPL32, RPS16, UCP2, HMOX1, RAP1A, VTN, MYC, TGFB1	4.189792	0.269627

GO:2000249	Regulation of actin cytoskeleton reorganization	8	0.384061	1.64E-04	PTK2B, RHOD, PHPT1, DAPK3, CD2AP, TGFB1, CSF1R, ARHGDIB	5.891102	0.30829
GO:0002376	Immune system process	57	2.736438	2.26E-04	H2-D1, TLR2, TLR4, C1QC, ISG20, B2M, CFP, NLRC5, TMEM173, H2-K1, PIK3CG, ADGRE1, BST2, HERC6, SERPING1, PRKCB, C1QA, C1QB, H2-AA, AKAP8, SSC5D, CD300LD3, IFITM2, C3, IFITM3, IL4RA, UNC93B1, RSAD2, IFI30, CD74, SRC, MIF, ALCAM, DDX3X, SQSTM1, PTK2B, TAP1, NRROS, CSF1R, MYO1G, H2-AB1, H2-Q7, FCGR1, PSMB8, LGALS9, IRF5, OTUB1, CD300A, IRF7, H2-EB1, LIME1, H2-T23, TAPBPL, H2-DMA, ZBTB1, THEMIS2, CD14	1.643894	0.424055
GO:0042590	Antigen processing and presentation of exogenous peptide antigen via MHC class I	5	0.240038	3.10E-04	H2-K1, IFI30, FCER1G, FCGR1, FCGR3	11.04582	0.581119
GO:0017145	Stem cell division	6	0.288046	0.0010298	FUBP1, CUL3, TIAL1, PAFAH1B1, THOC2, SAMD9L	6.627489	1.919222
GO:0006376	Mrna splice site selection	7	0.336054	0.001327031	SRSF10, LUC7L2, YTHDC1, MBNL1, PTBP2, LUC7L3, SETX	5.154714	2.466649
GO:0051028	Mrna transport	18	0.864138	0.002086036	ZFP36, NXT1, ENY2, SRSF1, XPO1, HNRNPA2B1, FMR1, EIF5A, IWS1, QK, HNRNPA3, SRSF7, FYTTD1, RANBP2, THOC2, TPR, NUP43, EIF5A2	2.259371	3.851451

GO:0048002	Antigen processing and presentation of peptide antigen	4	0.192031	0.002757279	SLC11A1, H2-AA, H2-AB1, CTSS	11.04582	5.060603
GO:0006915	Apoptotic process	72	3.456553	0.003439622	MEF2C, ITGB3BP, MEF2A, HRAS, HTATIP2, NUAK2, RBM5, EIF5A, RPS6KB1, PTEN, CTNNBL1, GPX1, BAK1, G2E3, TMEM173, USP53, SLK, CASP8AP2, TIA1, HMOX1, SEMA3A, DAP, CIB1, CCAR1, EBAG9, SGK1, C5AR1, ROCK1, DFFB, PKN2, CST3, DAPK3, MIEN1, STK3, DNASE2A, PURA, PRKCB, SLTM, TIAL1, MAPK3, TRIM39, C1D, BCLAF1, LITAF, ERBB4, XIAP, GULP1, EGLN3, TMEM219, CAAP1, DDX3X, SQSTM1, BCAP29, TRP53INP1, GAPDH, RBM25, PDCD10, TAOK1, LGALS1, TGFBR1, CIDEB, MALT1, GAS2, STAT1, SIRT1, NAE1, PRELID1, EPHA7, RASSF5, RABEP1, RNF152, PPID	1.395261	6.274981
GO:0045576	Mast cell activation	6	0.288046	0.004174452	FYB, FCGR2B, FCER1G, TLR4, FCGR3, LCP2	5.098069	7.566319

GO:0007049	Cell cycle	76	3.648584	0.004411565	ITGB3BP, CCNT2, E2F2, LZTS1, RPS6KB1, CD2AP, LATS1, CCNE2, CASP8AP2, EVI5, H2AFX, TPR, USP16, STAG2, STAG1, CIB1, CCAR1, ESCO1, BRCC3, PKN2, DYNLT3, MCM2, LIG4, MCM3, PPP1CB, MCM5, RBBP8, CCND1, PPP1CA, SASS6, UHRF1, RIF1, MAPK3, CDK2AP1, MAD2L2, PDCD2L, MAPRE3, NUP43, HAUS3, MPLKIP, GNAI3, 2610002M06RIK, NEK1, CETN3, LRRCC1, TXLNG, ANAPC10, CDC73, CNTRL, CCNG1, SRC, SPC24, WAPL, NIPBL, RB1CC1, PAFAH1B1, TERF1, TXNL4B, PDS5B, NASP, SMC5, BRCA2, GAS2, RACGAP1, APPL1, TET2, SMC2, SMC3, SMC4, NAE1, CDC25B, RPS6KA3, DMTF1, UBA3, BABAM1, CUL4B	1.367234	7.979397
GO:0043524	Negative regulation of neuron apoptotic process	26	1.2482	0.004876837	MEF2C, HRAS, XIAP, LGMN, CPEB4, UBE2V2, BDNF, KRAS, PTK2B, APOE, AGT, HMOX1, PIK3CA, MT1, ANGPT1, RASA1, OXR1, FZD9, C5AR1, ROCK1, BRAF, LIG4, SIRT1, CORO1A, CHL1, NGF	1.794945	8.784879

GO:0030335	Positive regulation of cell migration	31	1.488238	0.005066251	HMGB1, C3AR1, HRAS, ERBB4, PREX1, FERMT3, CX3CL1, TGFB1, ARHGAP5, PTK2B, SEMA3F, SEMA3D, SEMA3C, PDGFD, RHOD, CSF1R, CIB1, CCAR1, APC, PDCD10, TGFBR1, IGF1, MYO1F, SNAI2, DAPK3, MIEN1, CORO1A, CXCL16, MAPK8, WNT11, COL1A1	1.686799	9.110879
GO:0048511	Rhythmic process	22	1.056169	0.005343436	SRRD, ROCK2, UBE3A, CREB1, MTA1, CBX3, RORA, DDX5, SIRT1, PPP1CB, HNRNPU, SETX, TOP1, PPP1CA, HDAC2, NR1D2, HNRNPD, PSPC1, MAPK8, PRKAA2, CLOCK, FBXL3	1.8985	9.585952
GO:0032760	Positive regulation of tumor necrosis factor production	13	0.6241	0.00587419	HMGB1, CYBA, ZBTB20, HDAC2, TLR2, FCER1G, TLR4, H2-T23, PF4, NCL, CD14, PTAFR, LGALS9	2.433824	10.48907
GO:0051279	Regulation of release of sequestered calcium ion into cytosol	6	0.288046	0.00601477	CYBA, PTPN6, CORO1A, PTK2B, LIME1, NGF	4.733921	10.72684
GO:0045821	Positive regulation of glycolytic process	6	0.288046	0.00601477	GPD1, P2RX7, IGF1, PRKAA2, MYC, MIF	4.733921	10.72684
GO:0042752	Regulation of circadian rhythm	12	0.576092	0.006024081	PPP1CA, NR1D2, UBE3A, ROCK2, CREB1, PSPC1, HNRNPD, MAPK8, RORA, PRKAA2, PPP1CB, FBXL3	2.549034	10.74257
GO:0048147	Negative regulation of fibroblast proliferation	9	0.432069	0.00645226	CD300A, IFI30, TRP53INP1, CDC73, DACH1, EMD, MYC, FTH1, GSTP1	3.106636	11.46298
GO:0034341	Response to interferon-gamma	8	0.384061	0.006919256	SLC11A1, IRGM2, BST2, IFITM2, IFITM3, CXCL16, H2-EB1, H2-AA	3.398712	12.24242

GO:0000413	Protein peptidyl-prolyl isomerization	7	0.336054	0.006932046	PPIG, PPIA, PPID, PPIL4, RANBP2, NKTR, PPWD1	3.866035	12.26367
GO:0007568	Aging	27	1.296207	0.006962778	TSPO, LITAF, EIF5A, ITGB2, RPS6KB1, PTEN, TGFB1, ATP2B1, BAK1, APOD, APOE, DMD, AGT, P2RY1, CACYBP, NQO1, FGF2, CRYAB, CREB1, SERPING1, PPP1R9A, SERPINF1, ADM, UCP2, IFI27L2A, PEBP1, ADRA1D	1.723913	12.31472
GO:0071560	Cellular response to transforming growth factor beta stimulus	13	0.6241	0.007731058	MEF2C, TGFBR1, TGFB1, SRC, HDAC2, CLEC3B, CX3CR1, SERPINE1, COL1A1, SNRNP70, PDGFD, YES1, SCX	2.354026	13.58191
GO:0050731	Positive regulation of peptidyl-tyrosine phosphorylation	18	0.864138	0.008914625	CAV2, ICAM1, HCLS1, IGF1, VTN, TLR4, KITL, SRC, TGFB1, CD74, MIF, PTK2B, AGT, ANGPT1, YES1, RASA1, THBS4, GHR	1.968561	15.50019
GO:0060736	Prostate gland growth	5	0.240038	0.008979312	PLAG1, PRLR, UBE3A, IGF1, PTEN	5.522908	15.60387
GO:0016569	Covalent chromatin modification	37	1.776284	0.009552757	ENY2, BMI1, SMARCAD1, UTY, CBX3, DEK, NR3C1, HMGN5, CHD9, EPC2, MBTD1, CHD2, CHD1, PBRM1, PRKAA2, USP16, MYSM1, KMT5B, KDM7A, AEBP2, BRCC3, TET2, HLTF, DAPK3, UIMC1, PRKCB, ATRX, SUZ12, UHRF1, HDAC2, MTF2, ASH1L, BABAM1, SMARCA5, JMJD1C, HDAC9, SETD2	1.536448	16.51765
GO:0048286	Lung alveolus development	11	0.528084	0.009640061	ACE, PHF14, MAN1A2, ZFP157, PSEN2, BMPR2, MEG3, IGF1, RC3H2, TMTC3, FOXP2	2.531333	16.65595

GO:0001516	Prostaglandin biosynthetic process	6	0.288046	0.011266289	PNPLA8, PTGES2, TBXAS1, PTGDS, CD74, MIF	4.142181	19.19268
GO:0009617	Response to bacterium	9	0.432069	0.011331445	SLC11A1, P2RX7, FCGR2B, IRF8, TLR2, TLR4, STAT1, FCGR1, CD14	2.840353	19.29278
GO:0051726	Regulation of cell cycle	19	0.912146	0.011621118	E2F2, SGK1, TXLNG, INHA, CLIC1, CCNG1, PTEN, SRC, JUNB, CCNE2, BAK1, CCND1, SRSF5, TARDBP, UBA3, USP16, PLCB1, FGF2, APC	1.873844	19.73638
GO:0016525	Negative regulation of angiogenesis	14	0.672108	0.011778358	ROCK1, ROCK2, PF4, SPARC, SYNJ2BP, STAT1, SERPINF1, STAB1, KRIT1, AGT, CX3CR1, MEG3, TCF4, THBS4	2.147797	19.9762
GO:0000395	Mrna 5'-splice site recognition	4	0.192031	0.011994377	SRSF1, PSIP1, PRPF39, SNRPC	7.363877	20.30457
GO:0009408	Response to heat	12	0.576092	0.012147368	ALDOA, CKM, IGFBP7, PSIP1, HSPB2, TRP53INP1, PEBP1, IGF1, RPS6KB1, MAPK8, CASQ1, CD14	2.325435	20.53636
GO:0002474	Antigen processing and presentation of peptide antigen via MHC class I	9	0.432069	0.013442271	H2-K1, H2-Q4, H2-M3, H2-D1, H2-T22, H2-T23, H2-Q6, H2-Q7, B2M	2.761454	22.47279
GO:0048015	Phosphatidylinositol-mediated signaling	9	0.432069	0.013442271	PIK3CG, PIK3C2A, CAR8, IGF1, PIK3CA, NPR3, PTAFR, CSF1R, NGF	2.761454	22.47279
GO:0042542	Response to hydrogen peroxide	12	0.576092	0.013799356	GPX1, BAK1, PDCD10, PTK2B, CRYAB, HMOX1, MAPK8, COL1A1, SLC4A1, STAT1, SIRT1, SRC	2.285341	22.99887
GO:0006754	ATP biosynthetic process	7	0.336054	0.014284606	ALDOA, ATP5D, ATP5E, UQCC3, CHCHD10, ATP5O, TGFB1	3.36177	23.70836
GO:0036120	Cellular response to platelet-derived	7	0.336054	0.014284606	CREB1, PDGFD, SNAI2, YES1, MYC, SRC, RASA1	3.36177	23.70836

	growth factor stimulus						
GO:0048839	Inner ear development	13	0.6241	0.014392428	CEBPA, H2-K1, C1QB, BDNF, LRP10, CEBPD, IGFBP7, IGF1, SLC25A27, H2-T23, SPARC, H2-DMA, TGFB1	2.175691	23.86517
GO:0033138	Positive regulation of peptidyl-serine phosphorylation	14	0.672108	0.014701511	PHIP, BDNF, PDCD10, BRAF, HCLS1, AGT, PIK3CA, ANGPT1, AKAP9, MAD2L2, LATS1, TGFB1, FNIP1, MIF	2.089749	24.31297
GO:0060047	Heart contraction	6	0.288046	0.014793152	TRDN, GPX1, ACE, DNM1L, KCNE1, SGCD	3.898523	24.44527
GO:0002479	Antigen processing and presentation of exogenous peptide antigen via MHC class I, TAP-dependent	8	0.384061	0.015582868	H2-K1, PSMB3, PSMB2, H2-D1, H2-T23, H2-Q7, PSMB8, B2M	2.945551	25.57625
GO:0001937	Negative regulation of endothelial cell proliferation	8	0.384061	0.015582868	CAV2, APOE, TGFBR1, KRIT1, ATPIF1, SYNJ2BP, SPARC, STAT1	2.945551	25.57625
GO:0051602	Response to electrical stimulus	8	0.384061	0.015582868	P2RX7, HNRNPD, PEBP1, FZD3, AKAP9, SRC, S100A13, CD14	2.945551	25.57625
GO:0030501	Positive regulation of bone mineralization	9	0.432069	0.015826216	MEF2C, FZD9, ACVR2A, P2RX7, ISG15, BMPR2, CD276, GPM6B, TGFB1	2.68682	25.92152

GO:0000122	Negative regulation of transcription from RNA polymerase II promoter	84	4.032645	0.016435954	BMI1, MEF2C, CREBRF, NKAP, COPS2, XPO1, MEF2A, AEBP1, IMPACT, PRRX1, SPI1, ZEB2, ZKSCAN3, RLIM, TGFB1, CUL3, N4BP2L2, ZFP932, TPR, MYC, S100A1, ZFP36, ZBTB20, HIST1H1D, HIST1H1C, ZHX1, HNRNPA2B1, GABPA, JUNB, SUZ12, CCND1, UHRF1, MTF2, RPS14, PSEN2, MDM4, MAD2L2, LCOR, FRK, MTDH, ZBTB10, MYEF2, HIST2H3C2, CDC73, ZBTB18, NIPBL, CIR1, SQSTM1, SKIL, TCF4, CEBPA, HIST2H3B, AEBP2, PTPN2, HCLS1, TAF7, DACH1, SUPT4A, ZFP148, STAT1, DDX5, SNAI2, SIRT1, FNIP1, GCFC2, DUBR, FOXP2, ZFP748, IFI27, HDAC2, PHF14, RNF2, PPID, TRPS1, IRF8, HIST1H3B, ZFP281, HIST1H3C, HIST1H3D, HIST1H3E, HDAC9, HIST1H3F, ZBTB1, NFIB	1.272769	26.77998
GO:0006270	DNA replication initiation	7	0.336054	0.017622293	CCNE2, SLF1, POLA1, ORC4, MCM2, MCM3, MCM5	3.221696	28.42332
GO:0034375	High-density lipoprotein particle remodeling	5	0.240038	0.018280522	APOE, SCARB1, ABCG1, PLTP, ABCA5	4.602423	29.31999
GO:0051276	Chromosome organization	9	0.432069	0.018501153	GM7535, BRCA2, ATR, LIG4, MYC, SMC2, SMC3, SMC4, APC	2.616114	29.61816
GO:0051491	Positive regulation of filopodium assembly	8	0.384061	0.018597346	DNM3, GPM6A, FNBP1L, TGFBR1, FMR1, FSCN1, CCL21A, MIEN1	2.850533	29.74778

GO:0006974	Cellular response to DNA damage stimulus	52	2.496399	0.018708139	SMARCAD1, ERCC6L2, NEK1, SLF1, EGLN3, RPS27L, RRM2B, SETX, EPC2, NIPBL, ERCC6, CHD2, H2AFX, USP16, INO80D, FANCA, MYC, CIB1, APC, BOD1L, ALKBH7, ALKBH8, SGK1, SHPRH, BRCC3, TAOK1, FMR1, SMC5, SMC6, BRCA2, LIG4, ATR, SIRT1, SMC3, UIMC1, RBBP8, ATRX, CCND1, UVSSA, UHRF1, RIF1, ASCC3, OTUB1, TDP2, CDKN2AIP, MAPK3, BABAM1, CUL4B, MAD2L2, ZBTB1, CLOCK, REV3L	1.367577	29.8968
GO:0032355	Response to estradiol	17	0.816131	0.018979093	CRYAB, STRN3, CST3, PTGFR, PTEN, FCGR1, TGFB1, RBBP8, IFI27, CCND1, GRN, TFPI, WFDC1, COL1A1, NQO1, MYC, GSTP1	1.859197	30.25997
GO:0032496	Response to lipopolysaccharide	28	1.344215	0.019374494	ALDOA, ALAD, LITAF, CXCL9, TLR2, RPS6KB1, PF4, TLR4, SLC11A1, ACE, IL10RA, LOXL1, CHUK, C5AR1, ERBIN, MTA1, SPARC, STAT1, PTGFR, JUNB, LGALS9, RPS6KA3, P2RX7, HDAC2, ADM, RELT, MAPK3, PTAFR	1.569964	30.78676
GO:0071229	Cellular response to acid chemical	4	0.192031	0.019589	BDNF, S100A10, ENO1, NGF	6.311895	31.07096
GO:0044794	Positive regulation by host of viral process	4	0.192031	0.019589	CAV2, APOE, CFL1, ZFP72	6.311895	31.07096
GO:0046324	Regulation of glucose import	4	0.192031	0.019589	SLC25A27, RPS6KB1, APPL1, RTN2	6.311895	31.07096

GO:0060509	Type I pneumocyte differentiation	4	0.192031	0.019589	CREB1, IGF1, KLF2, NFIB	6.311895	31.07096
GO:0000165	MAPK cascade	13	0.6241	0.020083008	ZFP36, MEF2C, SLC11A1, DOK1, MEF2A, BRAF, PTK2B, MAPK3, PEBP1, PLVAP, MYC, TGFB1, SETX	2.081096	31.72129
GO:0000381	Regulation of alternative mrna splicing, via spliceosome	9	0.432069	0.021484269	MALAT1, SREK1, FMR1, RBM5, MBNL2, MBNL1, DDX5, NSRP1, RBM25	2.549034	33.53448
GO:0019882	Antigen processing and presentation	11	0.528084	0.021559226	H2-K1, H2-EB1, H2-D1, UNC93B1, H2-AA, FCGRT, H2-AB1, CTSS, H2-DMA, CD74, PSMB8	2.250074	33.63018
GO:0043406	Positive regulation of MAP kinase activity	11	0.528084	0.021559226	MEF2C, PIK3CG, HRAS, KRAS, PDCD10, PDGFD, FGF2, KITL, SRC, TGFB1, MIF	2.250074	33.63018
GO:0098792	Xenophagy	17	0.816131	0.022500388	HIST2H3B, HIST2H3C2, STOM, SLC35C1, SLC1A3, HIST1H3A, HIST1H3B, PNPO, HIST1H3C, HIST1H3D, HIST1H3E, ZFP189, HIST1H3F, YIPF1, HIST1H3H, CAR7, HIST1H3I	1.823096	34.8207
GO:1900095	Regulation of dosage compensation by inactivation of X chromosome	3	0.144023	0.023065634	JPX, FTX, RLIM	11.04582	35.52595
GO:0000398	Mrna splicing, via spliceosome	18	0.864138	0.0234781	TXNL4B, RBM41, LSM8, SRSF10, SNRPN, TRA2A, HNRNPA2B1, YTHDC1, HNRNPM, HTATSF1, RSRC1, PSPC1, SNRPC, RNPC3, SNRNP70, CWC22, NOVA1, PRPF40A	1.77522	36.03601

GO:0001916	Positive regulation of T cell mediated cytotoxicity	6	0.288046	0.023896151	H2-K1, P2RX7, H2-M3, H2-D1, H2-T23, B2M	3.488152	36.54909
GO:0070932	Histone H3 deacetylation	5	0.240038	0.024549886	SMARCAD1, ATXN3, HDAC2, HDAC9, SIRT1	4.248391	37.34361
GO:0002237	Response to molecule of bacterial origin	5	0.240038	0.024549886	H2-M3, TLR2, MALT1, CD14, B2M	4.248391	37.34361
GO:0048661	Positive regulation of smooth muscle cell proliferation	14	0.672108	0.026825088	RBPMS2, C3AR1, IGF1, RPS6KB1, CX3CL1, STAT1, CYBA, HMOX1, RAB5A, PDGFD, CALCRL, MYC, FGF2, PTAFR	1.933018	40.03598
GO:0010977	Negative regulation of neuron projection development	12	0.576092	0.027479229	H2-K1, HDAC2, LGALS1, DLEU2, RUNX1T1, H2-D1, PAFAH1B1, SEMA3A, THOC2, ITM2C, B2M, CIB1	2.07109	40.78955
GO:0051897	Positive regulation of protein kinase B signaling	16	0.768123	0.027556425	PIK3CG, MTDH, OSBPL8, HCLS1, TGFBR1, CCL21A, IGF1, ARFGEF1, SRC, STK3, TGFB1, GPX1, ITGB1BP1, ANGPT1, FGF2, RASD2	1.82199	40.87789
GO:0010764	Negative regulation of fibroblast migration	4	0.192031	0.029260874	BRAF, ITGB1BP1, CYGB, FGF2	5.522908	42.7968
GO:0006474	N-terminal protein amino acid acetylation	4	0.192031	0.029260874	NAA30, NAA50, NAA15, NAA16	5.522908	42.7968
GO:0016584	Nucleosome positioning	4	0.192031	0.029260874	HIST1H1D, RSF1, HIST1H1C, SMARCA5	5.522908	42.7968
GO:0045591	Positive regulation of regulatory T cell differentiation	4	0.192031	0.029260874	H2-M3, CD46, TGFB1, LGALS9	5.522908	42.7968
GO:0001798	Positive regulation of type iia hypersensitivity	4	0.192031	0.029260874	C3, FCER1G, FCGR1, FCGR3	5.522908	42.7968

GO:0007289	Spermatid nucleus differentiation	4	0.192031	0.029260874	GOPC, PYGO1, NECTIN2, TMF1	5.522908	42.7968
GO:0051301	Cell division	46	2.208353	0.029645343	CCNT2, ITGB3BP, HAUS3, GNAI3, MPLKIP, 2610002M06RIK, NEK1, LRRCC1, CETN3, ANAPC10, CNTRL, CCNG1, CD2AP, LATS1, SPC24, CCNE2, WAPL, EVI5, PAFAH1B1, TPR, USP16, STAG2, CIB1, TERF1, STAG1, PDS5B, BRCC3, PKN2, DYNLT3, SMC5, LIG4, RACGAP1, PPP1CB, CDC27, SMC2, MCM5, SMC3, SMC4, CDC25B, RBBP8, CCND1, PPP1CA, BABAM1, MAD2L2, NUP43, MAPRE3	1.358576	43.22142
GO:0048146	Positive regulation of fibroblast proliferation	12	0.576092	0.030469722	WAPL, AGT, BTC, SERPINE1, IGF1, PDGFD, LIG4, MYC, CD74, TGFB1, RASA1, MIF	2.039227	44.12184
GO:0045737	Positive regulation of cyclin-dependent protein serine/threonine kinase activity	7	0.336054	0.030731844	CCNT2, CCND1, CCNL1, CCNC, MAPRE3, SRC, CCNL2	2.86373	44.4053
GO:0070328	Triglyceride homeostasis	7	0.336054	0.030731844	MIA2, APOE, SCARB1, RORA, MED13, GPIHBP1, ANGPTL4	2.86373	44.4053
GO:0051090	Regulation of sequence-specific DNA binding transcription factor activity	5	0.240038	0.031963933	E130311K13RIK, CREBZF, HMOX1, MAPK3, FANCA	3.944934	45.71954
GO:0046685	Response to arsenic-containing substance	5	0.240038	0.031963933	ALAD, SERPINF1, SLC4A1, NR3C1, PTEN	3.944934	45.71954

GO:0070374	Positive regulation of ERK1 and ERK2 cascade	26	1.2482	0.032882635	HMGB1, HRAS, ERBB4, C3, TLR2, TLR4, CX3CL1, PTEN, TGFB1, CD74, SRC, MIF, GLIPR2, PTK2B, P2RY1, ANGPT1, PDGFD, FGF2, CSF1R, CIB1, ICAM1, C5AR1, BRAF, CCL21A, MAPK3, RAP1A	1.527613	46.68029
GO:0001558	Regulation of cell growth	11	0.528084	0.033931518	SGK1, WISP2, SGK3, HTRA1, IGFBP7, CSF2RB, WFDC1, CDC73, MAD2L2, IGFBP4, IL3RA	2.094896	47.75749
GO:0009611	Response to wounding	11	0.528084	0.033931518	ZFP36, GPX1, KLK8, SRSF5, SLC1A3, ADM, PEBP1, RPS6KB1, MYC, TGFB1, LCP1	2.094896	47.75749
GO:0045471	Response to ethanol	18	0.864138	0.034213922	ICAM1, ALAD, TBXAS1, G6PDX, TLR4, RPS6KB1, SPARC, PTEN, SIRT1, BAK1, CCND1, PTK2B, PEBP1, GK, NQO1, MYC, GSTP1, CD14	1.699356	48.04398
GO:0043393	Regulation of protein binding	6	0.288046	0.035980954	CYLD, TGFBR1, PSEN2, ANGPT1, ATR, SRC	3.155947	49.80307
GO:0000302	Response to reactive oxygen species	6	0.288046	0.035980954	GPX1, APOD, ATOX1, PTPRN, SOD3, GSTP1	3.155947	49.80307
GO:0090023	Positive regulation of neutrophil chemotaxis	7	0.336054	0.036216563	C3AR1, C5AR1, RAC2, CCL21A, NCKAP1L, CD74, THBS4	2.761454	50.03332
GO:0071407	Cellular response to organic cyclic compound	13	0.6241	0.036230579	MEF2C, CEBPA, CYBA, P2RY6, P2RX7, TMEM173, LGALS1, P2RY1, RAP1A, RPS6KB1, STAT1, TGFB1, TMF1	1.914608	50.04698

GO:0009615	Response to virus	14	0.672108	0.038210113	MEF2C, BST2, CREBZF, DDX3X, IFITM2, IFITM3, IFI27L2A, CFL1, RSAD2, DHX36, OAS1A, STMN1, SRC, ISG20	1.840969	51.94184
GO:0007229	Integrin-mediated signaling pathway	15	0.720115	0.039720647	ITGB3BP, ITGAL, PLP1, FERMT3, ITGB5, ITGB2, SRC, ITGAM, ADAM4, CUL3, PTK2B, ITGB1BP1, FCER1G, TYROBP, CIB1	1.781583	53.34177
GO:0071364	Cellular response to epidermal growth factor stimulus	8	0.384061	0.039851522	ZFP36, ERBB4, COL1A1, SNAI2, MYC, NCL, RASA1, GSTP1	2.454626	53.46123
GO:0031122	Cytoplasmic microtubule organization	8	0.384061	0.039851522	TRDN, SLK, CEP126, PCM1, ATAD1, HOOK3, APC, CIB1	2.454626	53.46123
GO:0043149	Stress fiber assembly	5	0.240038	0.040547676	CUL3, PHACTR1, PTK2B, ITGB5, SRC	3.681939	54.09179
GO:0044406	Adhesion of symbiont to host	4	0.192031	0.040991557	ICAM1, GBP7, NECTIN2, SCARB1	4.909251	54.48962
GO:0002504	Antigen processing and presentation of peptide or polysaccharide antigen via MHC class II	4	0.192031	0.040991557	H2-EB1, H2-AA, H2-AB1, H2-DMA	4.909251	54.48962
GO:0051451	Myoblast migration	4	0.192031	0.040991557	ROCK1, ITGB1BP1, PLEKHO1, THBS4	4.909251	54.48962
GO:1901409	Positive regulation of phosphorylation of RNA polymerase II C-terminal domain	4	0.192031	0.040991557	CCNT2, CCNL1, CCNC, CCNL2	4.909251	54.48962

GO:0006782	Protoporphyrinogen IX biosynthetic process	4	0.192031	0.040991557	ALAD, HMBS, IREB2, UROD	4.909251	54.48962
GO:0050684	Regulation of mrna processing	4	0.192031	0.040991557	SLTM, IWS1, NOVA1, SAFB2	4.909251	54.48962
GO:0035456	Response to interferon-beta	4	0.192031	0.040991557	BST2, IFITM2, IFITM3, STAT1	4.909251	54.48962

GO:0045944	Positive regulation of transcription from RNA polymerase II promoter	107	5.136822	0.04113971	JPX, MEF2C, HRAS, MEF2A, TLR2, TLR4, RORA, TGFB1, SETX, NLRC5, PSIP1, DHX36, GABPA, MED13, PTPRN, JUNB, CARF, ZFP462, MTF2, MAPK3, PYGO1, HMGB1, BMPR2, ATF1, SLC11A1, NIPBL, DDX3X, P2RY1, TCF4, PLAG1, IKZF2, MAFB, TAF7, ATAD2, ZFP148, TET2, DDX5, PAGR1A, HDAC2, RFX3, KLF2, ATAD2B, CCNT2, ARID4A, ARID4B, PRRX1, SPI1, ZEB2, PCGF5, TMEM173, USP16, FGF2, MYC, SCX, CHUK, SSBP4, CCNL1, NCOA7, CCNC, HLTF, HMGA1, NCL, CCNL2, ACVR2A, INHBA, ITGB1BP1, NCK1, ASH1L, CLOCK, NDN, UBE3A, CDC73, PF4, PAXBP1, NR3C1, PHIP, CIR1, CCDC62, CEBPZ, PPP3CB, NFAT5, PPP1R12A, ARMCX3, ETV1, FLT3L, ZFP292, YES1, ETV5, MYSM1, CEBPA, NUCKS1, CEBPD, HCLS1, CREB1, CEBPG, IGF1, SUPT4A, STAT1, SIRT1, ATRX, RPS6KA3, RPS6KA1, IRF5, ATXN7, IRF7, SP3, NFIB	1.187841	54.62167
GO:0006325	Chromatin organization	9	0.432069	0.041556386	HDAC2, ZFP462, NUCKS1, GPX4, ATAD2, HDAC9, HMGN5, FTX, SIRT1	2.259371	54.99113

GO:0006909	Phagocytosis	10	0.480077	0.04202148	ITGAL, SLC11A1, CORO1A, GULP1, PLD4, IRF8, RAB5A, MYO1G, PIK3CA, ITGB2	2.124195	55.40015
GO:0043484	Regulation of RNA splicing	7	0.336054	0.042290775	ZFP326, CLK4, MBNL2, MBNL1, CLK1, SNRNP70, HNRNPH1	2.666231	55.63537
GO:0006470	Protein dephosphorylation	20	0.960154	0.042612187	PTPN6, PTPN18, PTPN2, PTPN4, STYX, PTPRN, ACP1, PTEN, PTPRO, PPP1CB, CDC25B, RNGTT, CTDSPL2, MTM1, PPP1CA, PPM1E, PPM1K, PPP1R12A, PPP3CB, PHPT1	1.600843	55.91457
GO:0016064	Immunoglobulin mediated immune response	6	0.288046	0.043206722	FCGR2B, IRF7, IL4RA, FCER1G, H2-DMA, CD74	3.012495	56.42665
GO:0048260	Positive regulation of receptor-mediated endocytosis	6	0.288046	0.043206722	SERPINE1, CCL21A, VTN, TRF, BICD1, B2M	3.012495	56.42665
GO:0044262	Cellular carbohydrate metabolic process	3	0.144023	0.043373988	RPE, TKFC, GLB1	8.284362	56.5697
GO:0097368	Establishment of Sertoli cell barrier	3	0.144023	0.043373988	ICAM1, ARID4A, ARID4B	8.284362	56.5697
GO:0070537	Histone H2A K63-linked deubiquitination	3	0.144023	0.043373988	BRCC3, USP16, UIMC1	8.284362	56.5697
GO:2001199	Negative regulation of dendritic cell differentiation	3	0.144023	0.043373988	H2-M3, TMEM176B, TMEM176A	8.284362	56.5697
GO:0001845	Phagolysosome assembly	3	0.144023	0.043373988	CORO1A, P2RX7, SRPX	8.284362	56.5697
GO:0045901	Positive regulation of translational elongation	3	0.144023	0.043373988	EIF5A, USP16, EIF5A2	8.284362	56.5697

GO:0001805	Positive regulation of type III hypersensitivity	3	0.144023	0.043373988	FCER1G, FCGR1, FCGR3	8.284362	56.5697
GO:0001934	Positive regulation of protein phosphorylation	25	1.200192	0.043664093	HMGB1, HRAS, C3, BMPR2, TGFB1, MIF, KRAS, SQSTM1, RB1CC1, P2RY1, FLT3L, FGF2, RASA1, CIB1, CSF1R, ROCK2, SIRT1, FNIP1, ACVR2A, EPHA7, CCND1, P2RX7, MAPK3, CDK2AP1, NGF	1.50079	56.81675
GO:0001822	Kidney development	19	0.912146	0.045315656	NEK1, TGFBR1, FBN1, RPGRIP1L, MME, MMP17, CENPF, RRM2B, TET2, ACE, ZFP950, SERPINF1, AGT, PYGO1, WNT11, ROBO2, CAR2, C1GALT1, APC	1.614388	58.19804
GO:0071549	Cellular response to dexamethasone stimulus	8	0.384061	0.045457427	BMI1, RPL32, SERPINF1, CBX3, RPS6KB1, NR3C1, HNRNPU, TGFB1	2.388284	58.31464
GO:0001889	Liver development	15	0.720115	0.04634604	CEBPA, UPF2, CEBPG, RPGRIP1L, ASNS, SEC63, VWF, CCND1, KRAS, HLX, SP3, RB1CC1, HNRNPD, MEG3, PIK3CA	1.744076	59.0385
GO:0002230	Positive regulation of defense response to virus by host	18	0.864138	0.048173651	HIST2H3B, HIST2H3C2, STOM, SLC35C1, TMEM173, SLC1A3, HIST1H3A, HIST1H3B, PNPO, HIST1H3C, HIST1H3D, HIST1H3E, ZFP189, HIST1H3F, YIPF1, HIST1H3H, CAR7, HIST1H3I	1.62971	60.49002

GO:0042493	Response to drug	41	1.968315	0.049534341	XPO1, ALAD, TSPO, SORD, RPS6KB1, PTEN, SRC, TGFB1, B2M, TOP1, BAK1, BDNF, SLC1A3, PLIN2, APOD, PTK2B, BCHE, BLOC1S3, CHUK, RASA1, TERF1, ICAM1, COX8B, CREB1, LGALS1, CST3, NCKAP1L, FZD3, ATR, STAT1, JUNB, PRKCB, INHBA, CYBA, CCND1, P2RX7, HDAC2, SMPD1, PEBP1, WFDC1, COL1A1	1.335925	61.53893
